# Acute phase response following pulmonary exposure to soluble and insoluble metal oxide nanomaterials in mice

**DOI:** 10.1186/s12989-023-00514-0

**Published:** 2023-01-17

**Authors:** Claudia Torero Gutierrez, Charis Loizides, Iosif Hafez, Anders Brostrøm, Henrik Wolff, Józef Szarek, Trine Berthing, Alicja Mortensen, Keld Alstrup Jensen, Martin Roursgaard, Anne Thoustrup Saber, Peter Møller, George Biskos, Ulla Vogel

**Affiliations:** 1 Section of Environmental Health, Department of Public Health, University of Copenhagen, Copenhagen, Denmark; 2 National Research Centre for the Working Environment, Copenhagen, Denmark; 3 Atmosphere and Climate Research Centre, The Cyprus Institute, Nicosia, Cyprus; 4 National Centre for Nano Fabrication and Characterization, Technical University of Denmark, Copenhagen, Denmark; 5 Finnish Institute of Occupational Health, Helsinki, Finland; 6 Department of Pathophysiology, Forensic Veterinary Medicine and Administration, University of Warmia and Mazury in Olsztyn, Olsztyn, Poland; 7 Faculty of Civil Engineering and Geosciences, Delft University of Technology, Delft, The Netherlands

**Keywords:** Metal oxide, Nanomaterial, Acute phase response, Serum amyloid a

## Abstract

**Background:**

Acute phase response (APR) is characterized by a change in concentration of different proteins, including C-reactive protein and serum amyloid A (SAA) that can be linked to both exposure to metal oxide nanomaterials and risk of cardiovascular diseases. In this study, we intratracheally exposed mice to ZnO, CuO, Al_2_O_3_, SnO_2_ and TiO_2_ and carbon black (Printex 90) nanomaterials with a wide range in phagolysosomal solubility. We subsequently assessed neutrophil numbers, protein and lactate dehydrogenase activity in bronchoalveolar lavage fluid, *Saa3* and *Saa1* mRNA levels in lung and liver tissue, respectively, and SAA3 and SAA1/2 in plasma. Endpoints were analyzed 1 and 28 days after exposure, including histopathology of lung and liver tissues.

**Results:**

All nanomaterials induced pulmonary inflammation after 1 day, and exposure to ZnO, CuO, SnO_2_, TiO_2_ and Printex 90 increased *Saa3* mRNA levels in lungs and *Saa1* mRNA levels in liver. Additionally, CuO, SnO_2_, TiO_2_ and Printex 90 increased plasma levels of SAA3 and SAA1/2. Acute phase response was predicted by deposited surface area for insoluble metal oxides, 1 and 28 days post-exposure.

**Conclusion:**

Soluble and insoluble metal oxides induced dose-dependent APR with different time dependency. Neutrophil influx, *Saa3* mRNA levels in lung tissue and plasma SAA3 levels correlated across all studied nanomaterials, suggesting that these endpoints can be used as biomarkers of acute phase response and cardiovascular disease risk following exposure to soluble and insoluble particles.

**Supplementary Information:**

The online version contains supplementary material available at 10.1186/s12989-023-00514-0.

## Background

Acute phase response (APR) is a systemic response to an inflammatory state, where the concentration of acute phase proteins in the blood is altered. Inflammatory states such as trauma, infection, burns and advanced cancer induce APR. Advantageous effects of APR include tissue repair and recognition of pathogens; however, the two most upregulated acute phase proteins, C-reactive protein (CRP) and serum amyloid A (SAA) are considered risk factors for cardiovascular disease (CVD) [1, 2].

SAA is a family of proteins, composed of four isoforms (SAA1 to SAA4); while mice express all SAA isoforms, humans do not express SAA3 [3]. It is suggested that SAA is incorporated into high density lipoproteins (HDL) for transporting phospholipids and cholesterol for cell repair, binding lipids from dead cells and removing them from inflammation sites [4].

Although SAA has been used as a biomarker for atherosclerosis, SAA also plays a causal role in CVD in humans [3, 5, 6]. Furthermore, in murine models of atheroscleorosis, Dong and colleagues [7] showed that injecting apolipoprotein E deficient mice (ApoE^−/−^) on a chow diet, with a lentiviral vector containing the coding sequence of the SAA1 gene, resulted in increased SAA protein levels and an enhancement of plaque formation in the aorta arch. It was later demonstrated that SAA3 is present in aortic lesion in ApoE^−/−^ mice deficient in SAA1 and SAA2, and that an injection with an adeno-associated virus expressing SAA3, resulted in a marked increase in area of aortic lesions, whereas inactivation of the three inducible SAA isoforms (*Saa1, Saa2* and *Saa3*) diminished plaque formation [8]. We have shown that repeated intratracheal instillations of SAA prompt an increase in plasma SAA3 levels, accompanied by a progress in aortic plaques in *ApoE*^−/−^ mice [9], indicating that SAA promotes plaque formation in murine models of atherosclerosis. SAA also promotes foam cell formation of macrophages in vitro [10].

We have previously proposed that APR induction might constitute a causal link between particle inhalation and the progression of CVD [11–13]. We have exposed mice pulmonary to a variety of nanomaterials (NMs), including TiO_2_, single-walled carbon nanotubes (SWCNT), multi-walled carbon nanotubes (MWCNT), nanoclays, graphene plates, Printex 90 and ZnO, leading to an increase in pulmonary inflammation, *Saa3* mRNA levels and SAA3 plasma levels in a dose-dependent manner [14–19]. We have previously demonstrated that the pulmonary APR to engineered NMs exposure involves differential expression of many acute phase genes in a dose dependent manner [11, 13] and that the pulmonary transcription of APR genes is accompanied by dose-dependent increases in protein levels of several acute phase proteins [20, 21]. *Saa3* transcription has a high dynamic range, and for this reason, we have used *Saa3* expression as a biomarker of a pulmonary APR to particle exposure.

Metal fume fever (MFF) is a syndrome observed after inhalation of fumes containing metal oxide particles; MFF symptoms include fever, chills, cough, myalgia, headaches and malaise [22]. It has been suggested that MFF might be the result of the release of pro-inflammatory cytokines and neutrophil activation [22]. Recently, inhalation of fumes containing metal oxide nanoparticles by human volunteers was shown to increase blood levels of SAA and CRP before the onset of MFF symptoms [23, 24], thus providing a link between inhalation of metal oxides and risk of cardiovascular disease [11, 13]. In a controlled study, Baumann et al. (2018) exposed volunteers to welding fumes containing ZnO, CuO or both, for a period of 6 h, and reported a significant increase in SAA blood levels 24 h after exposure, that correlated with CRP levels from a previous study. Monsé and colleagues (2018) observed dose-dependent increase of CRP and SAA blood levels in healthy volunteers, after being exposed to a range of ZnO concentrations for 4 h. Additionally, they observed a high correlation between CRP and SAA blood levels. Brand and colleagues (2019) found a dose–response relationship between the cumulative dose of brazing fumes containing zinc and copper and blood levels of CRP, as a marker of APR [25]. These controlled exposure studies show causal relationships between exposure to soluble metal oxides and dose-dependent induction of APR in human volunteers. To our knowledge, there are no controlled human exposure studies using insoluble metal oxides but insoluble metal oxides are known to be capable of inducing metal fume fever [22].

We hypothesize that exposure to soluble and insoluble metal oxide NMs could induce APR in mice, possibly with different potency and time dependency. In this study we therefore aimed to evaluate pulmonary inflammation and APR induction in mice, after intratracheal instillation of five different metal oxide NMs with different solubility. Furthermore, we aimed to supplement the present results with results from previous studies using metal oxide NMs, in order to assess the effects of dosed surface area and solubility on APR.

## Materials and methods

### Nanomaterials

We selected five metal oxide NMs (ZnO, CuO, Al_2_O_3_, SnO_2_ and TiO_2_) based on their presence in an occupational environment (f. ex. welding fumes, material synthesis and downstream use), difference in solubility, and probable current occupational exposure in Denmark.

The metal oxide NMs were produced by spark ablation as described in previously reported studies [26–28]. In brief, material was ablated from two metallic electrodes placed at a distance of a few millimeters axially next to each other. Electrode materials were ablated to produce vapour clouds by repeated microsecond sparks induced between the electrodes by an electric circuit. The resulting vapours were subsequently quenched by a synthetic air gas flow containing trace amounts of O_2_, and condensed to form atomic clusters and subsequently nanoparticles upon condensation and coagulation [26]. Different metal electrodes were used in order to produce the respective oxide NMs. The resulting nanoparticles were collected on filters following the method described by Kourmouli et al. [29].

Carbon black NM (Printex 90), was a kind gift from Degussa-Hüls (today Evonic, Germany). In this study, TiO_2_ and Printex 90 were used as benchmark materials to enable comparison with previous studies [17, 20, 30–35].

### Nanomaterials suspension and characterization

Dose levels for CuO, Al_2_O_3_ and SnO_2_ were selected based on pilot studies. Dose levels for ZnO, TiO_2_ and Printex 90 were set based on previous studies [14, 17, 30, 36–38]. In brief, one mouse (n = 1) was exposed by intratracheal instillation and observed for one day. If the dose was tolerated by the mouse without signs of toxicity or overt MFF (i.e. hunched position, lack of movement, pilo erection and breathing difficulty) or marked body weight loss (approximately 10%), the dose was selected as the high dose. In turn, the same procedure was repeated with three mice (n = 3), which were observed for three days, to monitor mice wellbeing and body weight recovery. The low dose was a three-fold dilution from the high dose. In the case of CuO, an additional higher dose was included after a pilot study.

All NMs were dispersed in a solution of 2% v/v C57BL/6 mouse serum in Nanopure-filtered water, and sonicated on ice for 16 min and 10% amplitude, using a Branson Sonifier S-450D (Branson Ultrasonics Corp., USA) as described by Hadrup et al. [39]. The vehicle solution without NMs for control exposure was also sonicated. The lower NM dose was obtained by diluting the suspensions three-fold and sonicating for an additional 4 min. In the case of TiO_2_ and Printex 90, only one dose was used, while with CuO, one higher dose level was included. Table 1 shows the final doses used in this study. For histological analyses, mice were only exposed to control and the highest dose levels.

**Table 1:** Exposure dose level

Exposure	Dose level
Control vehicle	0 µg/mouse (0 mg/mL)
ZnO	0.7 and 2 µg/mouse (0.014 and 0.04 mg/mL)
CuO	2, 6 and 12 µg/mouse (0.04, 0.12 and 0.24 mg/mL)
Al_2_O_3_	18 and 54 µg/mouse (0.36 and 1.08 mg/mL)
SnO_2_	54 and 162 µg/mouse (1.08 and 3.24 mg/mL)
TiO_2_	162 µg/mouse (3.24 mg/mL)
Printex 90	162 µg/mouse (3.24 mg/mL)

The hydrodynamic size distribution of the NM suspension was measured by Dynamic Light Scattering (DLS) using a Malvern Zetasizer Nano ZS (Malvern Instruments, UK). Results were given as the average of six consecutive measurements from the exposure suspension of each dose level (n = 1).

Transmission electron microscopy (TEM) analysis was conducted in order to characterize physical parameters of individual particles of the six NMs in exposure vehicle. Samples were prepared on TEM grids directly from the NM suspension prepared for instillation. The samples were prepared by drop casting 2 µL of suspension directly onto Ni TEM grids with a continuous carbon/Formvar film. The droplets were left on the grids for 10 s, before excess liquid was blotted away with a tissue.

The TEM analysis was made using a Tecnai T20 G2 (FEI Company, USA) equipped with a XF416 CCD camera (TVIPS GmbH, Germany). Samples were imaged at 200 keV, at varying magnifications in order to perform size measurements of both agglomerates, primary particles, and lattice spacing for crystal structure information. Agglomerate sizes were determined from low magnification images, using a manually-set global threshold in order to differentiate particles from the substrate. The number of agglomerates counted for each NMs were: 7920 particles for Al_2_O_3_, 8239 particles for CuO, 3438 particles for SnO_2_, 46 particles for TiO_2_, and 697 particles for ZnO. Primary particle sizes were determined with a watershed algorithm when possible, but most samples required manual measurements, where a circle was fitted to the individual nanoparticles. Primary particle size was measured on at least 15 primary particles from each sample. Lattice spacing was determined from at least 10 different particles for each sample via FFT analysis. All the analysis were conducted with either ImageJ or via custom Python scripts. Both agglomerate and primary particle sizes are reported as equivalent circular diameters.

The electrical mobility sizes of the agglomerated particles produced were measured online during spark ablation system using a Scanning Mobility Particle Sizer (SMPS), consisting of Differential Mobility Analyzer (TSI, Model 3085), and a Condensation Particle Counter (TSI Model 3025). The specific surface area of the metal oxide powders was analyzed using the BET nitrogen absorption method (3P sync 110). In brief, samples were dried at 200 ºC under vacuum for 2 h, weighed (with amounts ranging from 14 to 130 mg) and analyzed with the static volumetric method.

Solubility of Al_2_O_3_ and SnO_2_ NMs was determined by dissolution in phagolysosomal simulant fluid (PSF) (pH = 4.5), using an Atmosphere-Temperature-pH-controlled Stirred Batch Reactor (ATempH SBR) as described by Holmfred et al. [40]. NMs were first pre-wetted with 0.5% ethanol, followed by their dispersion in 0.05% BSA water to a concentration of 1.25 g/L and sonication, as described previously. NMs were then dispersed in PSF to a concentration of 50 mg/L, and incubated for 24 h and 37 ºC in the ATemph SBR. After incubation, 4 mL of supernatant were centrifuged for 30 min at 4400 rcf using 3 kDa centrifugal filters. The dissolved ions were stabilized with 2% HNO_3_, and the concentration of metal ions in the supernatant was measured by ICP-MS. Solubility in PSF of ZnO, CuO and TiO_2_ NMs was not quantified, as previous studies had already provided solubility values following the same procedure and PSF test media as applied in this study [41]. Table 2 presents values taken from literature. More information about the dissolution analysis is found in the Additional file 1.

**Table 2:** Physicochemical characteristics of NMs used

Name	Primary particle size (nm)^1^	Specific surface area (m^2^/g)	SMPS peak particle diameter (nm)^2^	Solubility in PSF (mg/L)	Z average size (nm)^1^	PDI^1^
ZnO	5.7 ± 3.8	15.2	88.6	> > 100^3^	151.1, at 0.04 mg/mL	0.31
CuO	7.8 ± 6.8	124.1	37.8	101.9 ± 10.4^4^	146.4, at 0.24 mg/mL	0.24
Al_2_O_3_	5.5 ± 5.5	275.9	81.9	0.311 ± 0.009	95.1, at 1.08 mg/mL	0.50
SnO_2_	4.5 ± 2.3	165.4	28.6	< LOD^5^	105.1, at 3.24 mg/mL	0.50
TiO_2_	6.6 ± 4.1	297.1	57.9	< LOD^3^	225, at 0.36 mg/mL	0.44
Printex 90	14^6^	295–338^6^	–	ND^7^	80 at 3.24 mg/mL	0.16

^1^Measured in exposure vehicle.

^2^Measured during spark ablation.

^3^LOD for TiO_2_: 0.0143 mg/L, [40].

^4^Unpublished results.

^5^LOD for SnO_2_: 0.00433 mg/L (Additional file 1).

^6^(Saber et al., 2019).

^7^Not determined (ND).

### Animals housing and exposure

Female C57BL/6 J mice of seven weeks of age (Janvier Labs, France) were randomly distributed into polypropylene cages according to the exposure and allowed to acclimatize for one week. Mice exposed to vehicle control were caged in groups of 3–4 animals, 6 animals in the case of NM exposure and 3 animals for histology. The study was carried it out staggered, therefore for organ endpoints (i.e. non-histopathology), we used n = 4–6 mice as vehicle control on every day we exposed mice to NMs, and n = 6 mice for each dose level of NMs exposure. In total, the combined vehicle control group was n = 26 for both post-exposure time points. In the case of histopathology, n = 3 for vehicle control and NM exposure mice. The cages contained Enviro-dri bedding (Brogaarden, Denmark), and wood blocks and hides as enrichment. Mice had access to food (Altromin 1324 M, Brogaarden, Denmark) and tap water ad libitum*.* Room temperature was set at 20 ± 2 ºC, while humidity were 50 ± 20%. Finally, mice were housed under 12 h cycles of light and dark.

For the vehicle (control) and NM exposure, mice were first anaesthetized by inhalation of 4% isoflurane. A volume of 50 µL of vehicle control or NM suspension was then intratracheally instilled once, followed by 150 µL of air [42]. Mice were observed after instillation to confirm that their airways were not blocked.

### Blood, BALF and tissue collection

At 1 or 28 days after NM exposure, mice were anaesthetized by an intraperitoneal injection of ZRF cocktail (Zoletil Forte 250 mg, Rompun 20 mg/mL and Fentanyl 50 µg/mL in sterile isotone saline) and killed by exsanguination by withdrawal of heart blood. Blood samples were mixed with K_2_EDTA and kept on ice. Plasma was obtained by centrifuging the blood samples for 10 min at 2500 rcf and 4 ºC [43].

Bronchoalveolar lavage fluid (BALF) was collected by flushing the lungs twice with 0.9% sodium chloride and kept on ice. Samples were centrifuged for 10 min at 400 rcf and 4 ºC [43]. The supernatant was collected for BAL protein content and lactate dehydrogenase (LDH) activity analysis, while the cell pellet was resuspended in Ham’s F-12 medium with 10% fetal bovine serum [32]. Mouse lung and liver tissue was harvested, sectioned, snap-frozen in liquid nitrogen and stored at − 80 ºC.

### BAL protein content and LDH levels in BALF

The protein content in BALF was measured using the Pierce BCA Protein Assay Kit (Cat. No. 23225, Thermo Fisher Scientific), following the manufacturer´s instructions. Each sample was analyzed in duplicates and protein concentration was obtained from the standard curve.

LDH activity in BALF was determined using a colorimetric LDH assay kit (Cat. No. ab102526, Abcam). Samples were treated in duplicates following the kit’s instructions and incubated for 30 min at 37 ºC. LDH activity was calculated using NADH concentrations obtained from the standard curve.

### Cell differential counting

Cells obtained from BALF were concentrated onto microscope slides using a Cytofuge 2 cytocentrifuge (VWR—Bie and Berntsen, Denmark) for 4 min at 60 g. Following centrifugation, slides were air dried for 30 min and fixed in 96% ethanol for 5 min. Cells were then stained for 3 min with May-Grünwald stain and for 30 min with Giemsa stain. Differential count was performed by counting and classifying 200 cells per sample, using light microscopy [43].

### Saa3 and Saa1 mRNA expression

We used *Saa3* as a biomarker for pulmonary APR as it was the most upregulated gene when exposing mice by inhalation to TiO_2_ NM, while *Saa1* was only slightly upregulated in lungs [44], but the most differentially regulated *Saa* isoform in liver [13, 20, 45–47]. We have previously shown that pulmonary inflammation assessed as neutrophil influx is correlated to the deposited surface area of NM, pulmonary *Saa3* mRNA levels and to plasma SAA3 protein levels [15, 16, 48].

*Saa3* mRNA levels were measured in lung tissue, while *Saa1* mRNA levels were measured in liver tissue. In both cases, RNA was purified from frozen tissue using Maxwell® 16 LEV simplyRNA tissue kit (Cat. No. AS1280, Promega) following the manufacturer’s instruction. RNA concentrations were measured using a Nanodrop 200c spectrophotometer (Thermo-Fisher Scientific, USA). Following this, complimentary DNA was synthetized with TaqMan™ Reverse Transcriptase reagents (Cat. No. N8080234, Thermo-Fisher Scientific). The expression of both genes was determined using a modified protocol for TaqMan™ Fast Advance Master Mix (Cat. No. 4444557, Thermo-Fisher Scientific). For *Saa3* determination, forward and reverse primers were from TAG Copenhagen and *Saa3* probe was from Thermo-Fisher Scientific (Cat. No. 450025). In the case of *Saa1,* a primer and probe mixture was used (Cat. No Mm00656927_g1, Thermo-Fisher Scientific). Samples were analyzed in triplicates, using the ViiA 7 Real-time PCR system (Thermo-Fisher Scientific) and 18S gene as reference (Cat. No. 4310893E, Thermo-Fisher Scientific) [16]. In the present study, we also analyzed *Saa3* mRNA levels in lung tissue from a previous study, where female C57BL/6 J mice of eight weeks of age were intratracheally instilled with 2, 6 or 18 µg of uncoated ZnO NM and tissue collected after 1 day [37].

### SAA3 and SAA1/2 plasma protein levels

Plasma levels of SAA3 were measured using ELISA from Merck Millipore (Mouse SAA-3, Cat. No. EZMSAA3-12 K), while plasma levels of SAA1/2 were determined using ELISA from Tridelta (PHASE™ Murine Serum Amyloid A Assay, Cat. No. P-802 M). The manufacturer’s instructions were followed for both assays and samples were measured in duplicates. In both cases, protein levels were measured only on samples from day 1 after NM exposure, as previous experience with the same assays showed no significant change in SAA3 and SAA1/2 plasma levels after 28 and 92 days post-exposure [16]. In addition, the same study showed that the assays did not present cross reactivity [16]. SAA3 plasma protein levels were also measured in samples from Jacobsen and colleagues (2015) study; these include plasma from female C57BL/6 J mice of eight weeks of age after 1 day of been exposed to 6 and 18 µg of uncoated ZnO NMs.

### Histopathology

After 1 or 28 days, mice were anaesthetized and exsanguinated through a cut in the femoral artery. Lungs were filled with 10% neutral buffered formalin with a content of 4% formaldehyde, using a 30 cm water column pressure. A knot was tied around the trachea to fix the lung in an inflated state [48, 49]. Lungs were then removed and set in 10% neutral buffered formalin, for at least 24 h. Liver samples were collected following the revised guides for organ sampling [50], and fixed in 10% neutral buffered formalin with a content of 4% formaldehyde.

Lungs and livers were processed using a Leica TP1020 tissue processor (Leica Biosystems), and paraffin embedded using a HistoCore Arcadia embedding center (Leica Biosystems). Sections were cut at 4–5 µm with a HistoCore Autocut rotary microtome (Leica Biosystems), introduced to a Leica HI1210 water bath at 40 ºC (Leica Biosystems) and placed on microscope slides. For staining, samples were first dried at 60 ºC for 1 h, and later stained with hematoxylin and eosin (H&E) or Sirius red. Brightfield images were acquired at 20 × and 40 × magnification on an Olympus BX 43 microscope with a 0.5 × C-mount and a Nikon DS-Fi2 camera.

Histological examination of livers was carried out on samples from animals exposed to vehicle control or high dose level of NMs, 28 days post-exposure (n = 3/group). Histological examination of the liver samples by light microscopy in brightfield mode was performed by two operators, first with knowledge of treatment groups and thereafter blindly [51]. The diagnostic terms followed the International Harmonization of Nomenclature and Diagnostic Criteria for Lesions in Rats and Mice (INHAND) proposal for diagnostic nomenclature of microscopic changes in hepatobiliary system of laboratory rodents [52]. Severity scores were given for the following changes if observed: mitosis, karyomegaly, karyocytomegaly, binucleate hepatocytes, apparent increase in Kupffer cells, Kupffer cells with prominent nuclei, hyperplasia of connective tissue near bile ductules and/or venules and hyperplasia of oval cells. The severity was evaluated using a 5-grade system: grade 1: minimal/very few/very small; grade 2: mild/few/small; grade 3: moderate/moderate number/moderate size; grade 4: marked/many/large; and grade 5: massive/extensive number/extensive size. Inflammatory cell infiltrates (focal infiltrations of mono- and polynuclear and/or histiocytic cells) were divided into two categories: small (≤ 10 inflammatory cells, sporadically accompanied by necrotic hepatocytes with distinct eosinophilic cytoplasm and/or presence of apoptotic bodies) and big (> 10 inflammatory cells often surrounded by necrotic hepatocytes with distinct eosinophilic cytoplasm, with apoptotic bodies/debris often present).

The lung tissues from vehicle control and high dose groups (n = 3/group) were evaluated using a structured scoring sheet for recording diverse histological changes as described earlier [17, 49]. For each scored entity, the scoring was performed on the lung lobe with the most histopathological changes. Lymphocytic infiltrates were defined as areas of tissue where the density of lymphocytes is higher than background so that the lymphocytes collection had a shape and size. These were usually present around bronchi and blood vessels. The minimum requirement was that they should contain 50 or more lymphocytes. Macrophage aggregates were defined as areas of tissue where the density of macrophages is higher than background so that the macrophages can be seen as group. The minimum requirement was that they should contain 5 or more macrophages. In addition, a number of other parameters was evaluated including the presence of free or phagocytosed foreign material, interstitial thickening, fibrosis, granuloma and pulmonary alveolar proteinosis.

### Statistics

All data were analyzed using GraphPad Prism 9 (GraphPad Software Inc., USA) and results are presented as mean ± standard deviation. Results from the control group were combined for each exposure time point. Additionally, one sample of BALF from control group for 28 days was lost due to technical errors.

All datasets, with the exception of total protein content in BALF, were log-transformed to fulfill normal distributions of residuals and variance homogeneity between exposure groups. The effect of the exposure on BALF cellularity, BALF protein content, BALF LDH activity, *Saa1* mRNA levels and SAA1/2 plasma protein level were calculated using one-way ANOVA, with a post-hoc Dunnett comparison test against the control group. In the case of *Saa3* mRNA levels and SAA3 plasma protein levels after NM exposure, where normal distributions and homogeneity of variance was not reached after log-transformation, Brown-Forsythe ANOVA test was used, with a post-hoc Dunnett T3 test. Results obtained during this study and previous studies were correlated using Pearson correlation coefficient.

## Results

### Particle characteristics

A summary of the physico-chemical characteristics of the studied NMs is shown in Table 2.

**Fig. 1:**
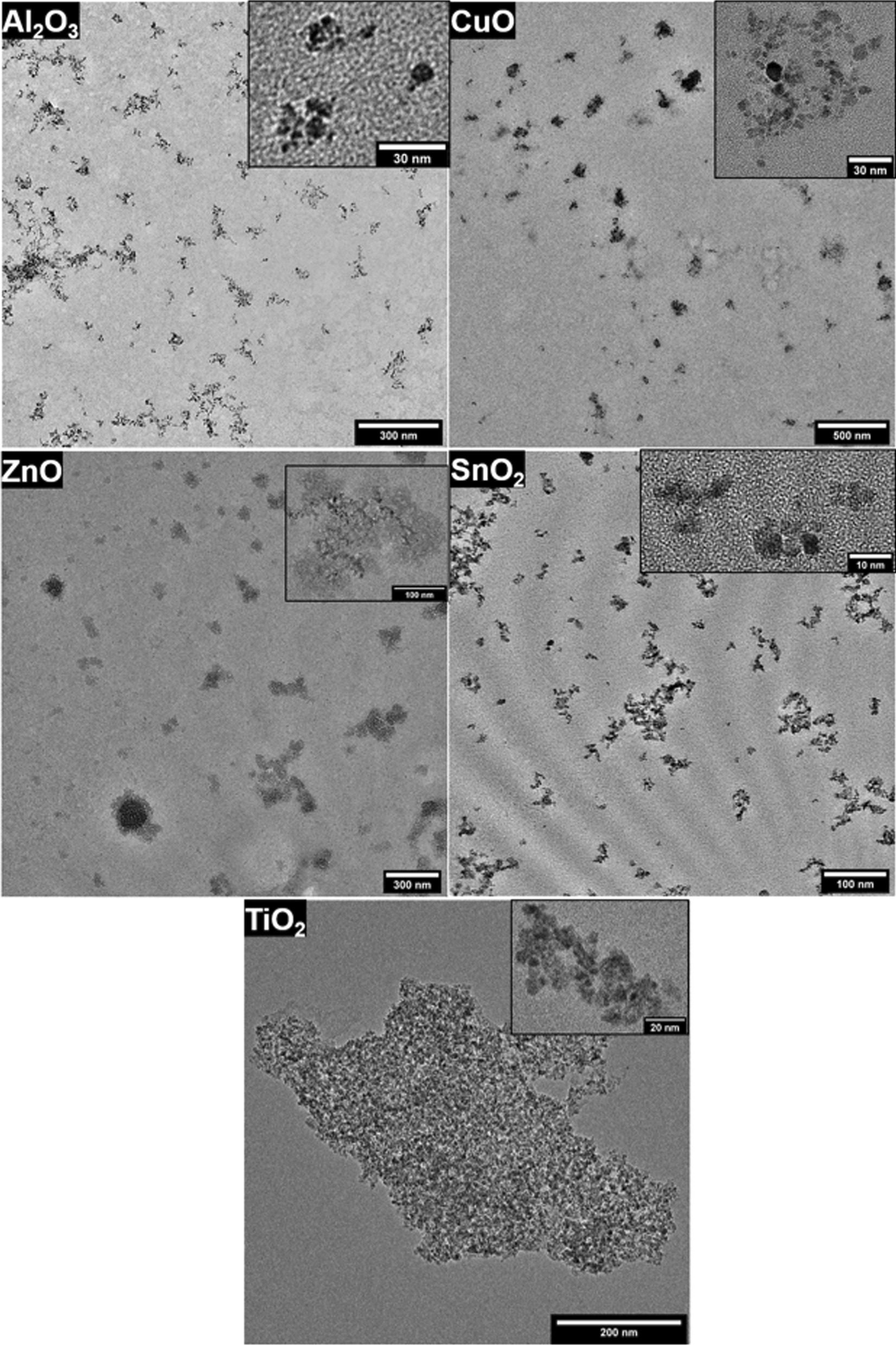
Representative TEM images of all metal oxides NM. An additional high magnification image of a single agglomerate is displayed in the top right of each image

Primary size and agglomerate size of particle in exposure vehicle was measured using TEM. Images from the electron microscopy analysis of each sample are shown in Fig. 1, while the particle diameter distributions are presented in the Additional file 2: Figure S1. We observed that agglomerate shapes and sizes varied significantly between different NMs. The primary particle sizes ranged from 4 to 8 nm. Lattice spacings were measured for all compounds, except Al_2_O_3_, where sample drift at the required high magnification made it difficult to acquire sharp images (Additional file 3). The measured lattice spacings were consistent with the expected nanoparticle crystal structures. Agglomerates of Al_2_O_3_ and SnO_2_ showed sizes of approximately 15 nm with a soot-like structure (i.e. forked chains of agglomerated nanoparticles). In contrast, CuO and ZnO agglomerates displayed denser structures with larger sizes of approximately 40 and 30 nm, respectively. TiO_2_ agglomerates were very few in number, presenting either in the 20 nm size range, or consisting of hundreds of primary spheres, measuring sizes larger than 200 nm. Findings from the TEM analysis are summarized in Additional file 3: Table S1.

The electrical mobility peak sizes of the agglomerated particles produced during spark ablation, prior to particle collection, were less than 100 nm for all metal oxides (Table 2). Results from the BET analysis showed specific NM surface areas that ranged from 15 to ca. 300 m^2^/g.

Dissolution of Al_2_O_3_ and SnO_2_ in PSF was determined using a ATempH SBR [40]. No dissolution of SnO_2_ was detected in PSF within the 24 h period of the analysis. In the case of Al_2_O_3_, the results show rapid dissolution and limited solubility of 0.311 mg Al_2_O_3_/L. Detailed information of dissolution profiles are found in the Additional file 1. For ZnO and TiO_2_, PSF solubility values of the same substances were tested in Holmfred et al. [40] using the same approach. For ZnO, the solubility limit was higher than 100 mg/L (used test concentration) and for TiO_2_ no dissolution was observed. For a CuO NM, the solubility was found to be 101.9 mg/L in PSF (unpublished results) and Printex 90 is considered insoluble in the same setup. It should be noted that solubility and rates may vary between materials, but the results from the tested materials are considered sufficiently indicative for the purpose of this paper. From the results, we define ZnO and CuO as to be highly soluble; Al_2_O_3_ to be intermediately soluble, while SnO_2_, TiO_2_ and Printex 90 are poorly to insoluble in PSF. However, for the purpose of this study, we have grouped ZnO and CuO as soluble NMs, and Al_2_O_3_, SnO_2_, TiO_2_ and Printex 90, as insoluble NMs.

The hydrodynamic size distribution of the NM in exposure vehicle was determined by Dynamic Light Scattering (DLS). The number-based size distribution (Additional file 4: Figure S2) showed that Al_2_O_3_, SnO_2_, TiO_2_ and Printex 90 presented unimodal peaks at 28 nm, 24 nm, 44 nm and 38 nm, respectively. ZnO and CuO suspensions showed bimodal distributions. For ZnO, we observed a major peak at 79 nm, and a minor peak at 12 nm; while for CuO, we observed two unresolved peaks, the major at 44 nm and the minor at 16 nm. The Z-average size varied between 80 and 225 nm, based on their DLS-measured intensity, while the polydispersity index (PDI) varied between 0.16 and 0.50 (Table 2).

### Histopathology analysis

**Fig. 2:**
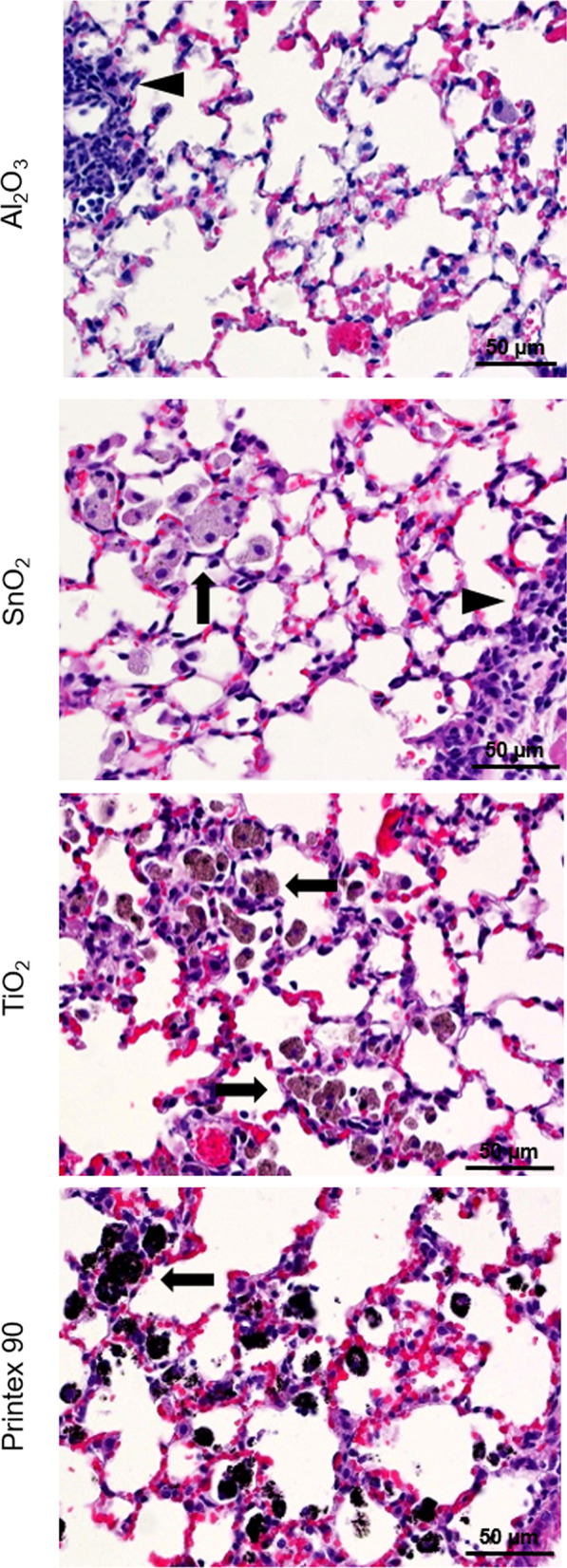
Mouse lung histopathology 28 days post-exposure to Al_2_O_3_, SnO_2_, TiO_2_ and Printex 90. Macrophage aggregates in the alveolar region (arrows) and perivascular lymphocytic infiltration (arrowheads). Brightfield microscopy, H&E stain, and scale bar (50 μm) applies to all

There were no histopathological changes in the lung tissue 1 and 28 days post-exposure to the highest dose of ZnO and CuO, and only few lymphocytic infiltrations after Al_2_O_3_ exposure (Fig. 2 and Additional file 5: Figure S3). No particles were observed for ZnO and CuO. For SnO_2_, TiO_2_ and Printex 90, macrophage aggregates and foreign material were seen in addition to lymphocytic infiltrations. The changes were most prominent after exposure to TiO_2_ and Printex 90 (Additional file 6: Table S2).

**Fig. 3:**
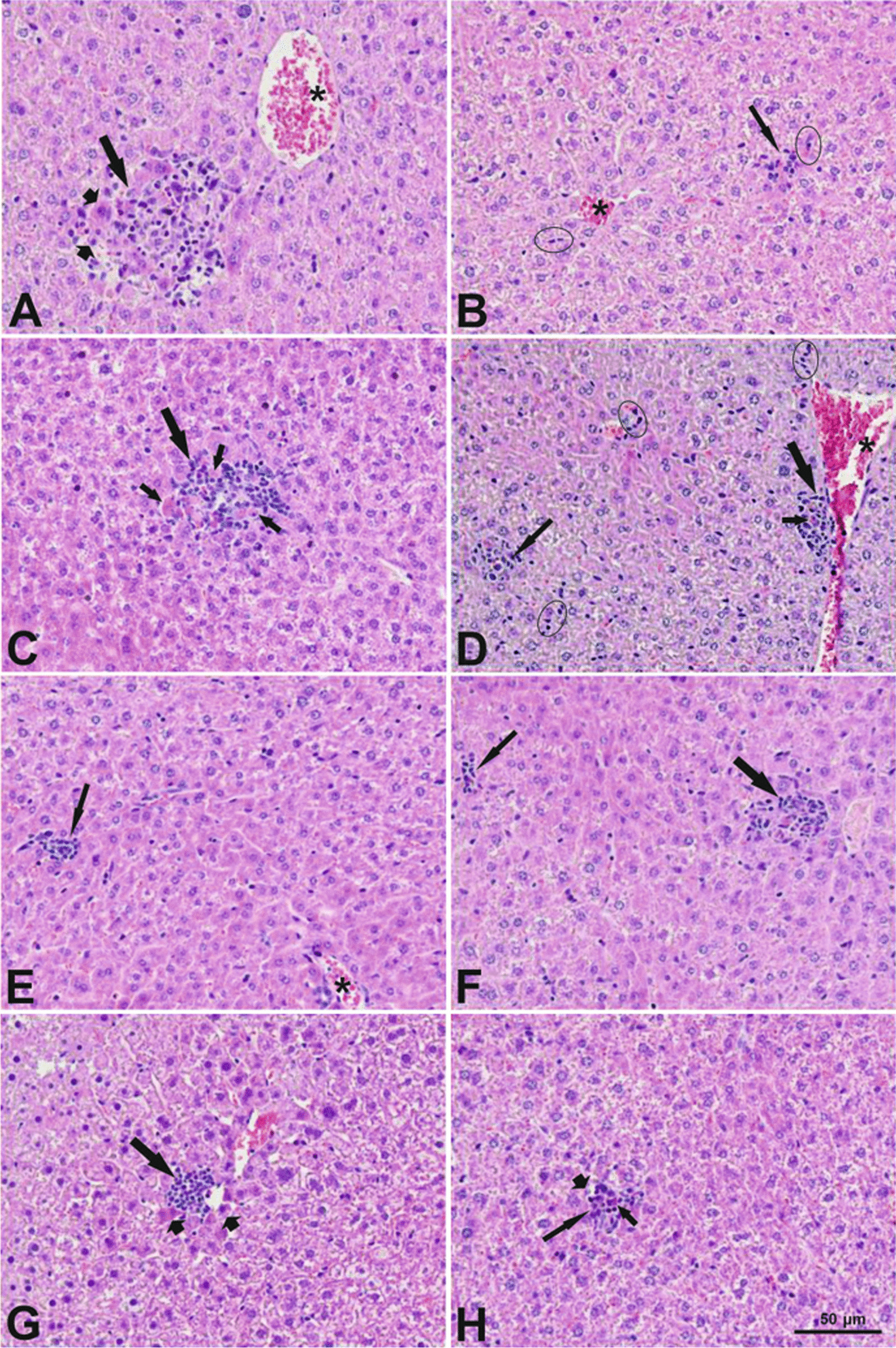
Examples of focal inflammatory cell infiltrates in livers of mice 28 days after intratracheal exposure to a single dose of vehicle control or NMs. Micrographs represent individual groups: **A** vehicle control, **B** ZnO 2 µg/animal, **C **CuO 6 µg/animal, **D** CuO 12 µg/animal, **E **Al_2_O_3_ 54 µg/animal, **F **SnO_2_ 162 µg/animal, **G** TiO_2_ 162 µg/animal, **H **Printex 90 162 µg/animal. Long arrows indicate a focal big inflammatory cell infiltration in **A**, **C**, **D**, **F** and **G**. Long thin arrows indicate a focal small inflammatory cell infiltration in **B**, **D**, **E**, **F** and **H**. Short thick arrows indicate necrotic hepatocytes in **A**, **G** and **H**. Short thin arrows indicate apoptotic bodies in focal inflammatory cell infiltrates in **C**, **D** and **H**. Ellipses indicate a local minimal increase of Kupffer cells in **B** and **D**. Asterisks indicate congestion in blood vessels in **A**, **B**, **D** and **E**. HE staining, magnifications were the same for all images as shown on image **H**

Several histological changes were seen in the livers of mice 28 days after intratracheal exposure to the vehicle control or NMs (Additional file 7: Table S3). Overall, the type, incidence and minimal severity of the changes indicated no difference between the vehicle control and the exposed groups in morphology of the liver, with the exception of inflammatory cell infiltrations. Although small and big inflammatory cell infiltrations were present in the vehicle control and all groups exposed to the NMs (Fig. 3), an elevated number of these infiltrations was recorded for CuO and SnO_2_ exposed animals (Additional file 7: Table S4). In case of CuO, the mean numbers of combined (big and small) and small inflammatory cell infiltrations were elevated at both dose levels, and big cell infiltrations at the highest dose. In case of SnO_2_, the numbers of combined small and big inflammatory cell infiltrations were elevated.

### BALF cell composition and protein levels

**Fig. 4:**
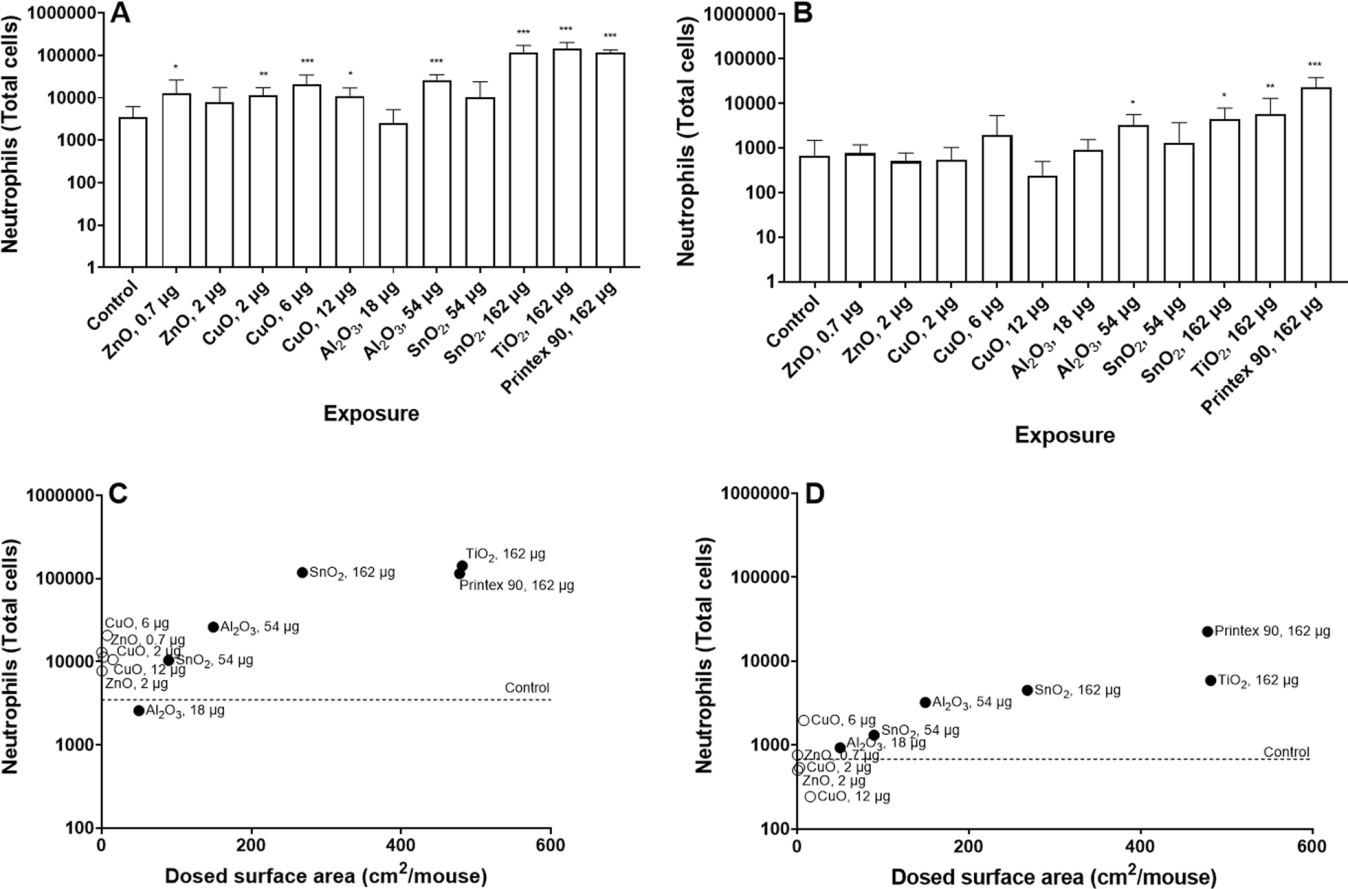
Neutrophil numbers in BAL fluid 1 (**A**) and 28 (**B**) after exposure to NM. Data are shown as mean and bars represent SD. *, ** and ***designate *p *values < 0.05, < 0.01 and < 0.001 respectively vs. control. Correlations between dosed surface area and neutrophil numbers, 1 (**C**) and 28 (**D**) days after exposure

To assess the pulmonary inflammatory response after exposure, we quantified the number of neutrophils in BALF (Fig. 4A and B). We found a statistically significant increase in neutrophil numbers in almost all exposure groups 1 day after exposure, with the exemption of the high dose level of ZnO and the low dose level of Al_2_O_3_ and SnO_2._ Following 28 days after exposure, the number of neutrophils decreased in all groups, however in mice exposed to Al_2_O_3_, SnO_2_, TiO_2_ and Printex 90, the neutrophil count was significantly elevated at the high dose level. As Al_2_O_3_ induced inflammation at day 28 even though it had an intermediate solubility, we have grouped it with the insoluble NMs. This suggests that pulmonary inflammation was induced by all NMs, but it was more pronounced after exposure to the poorly soluble SnO_2_, TiO_2_ and Printex 90, and remained induced for 28 days after exposure to insoluble NMs (i.e. Al_2_O_3_, SnO_2_, TiO_2_ and Printex 90), but not for soluble NMs (i.e. ZnO and CuO). Total BAL cell composition can be found in Additional file 8: Table S5.

When correlating neutrophil numbers with the exposure dose levels expressed as pulmonary deposited particle surface area, we found a statistically significant correlation to the dosed particle surface area after 1 and 28 days (day 1: r = 0.84, *p *value < 0.001; day 28: r = 0.86, *p *value < 0.001), as shown in Fig. 4C and D. When subdivided by solubility, the two soluble NM, CuO and ZnO, seemed to induce inflammation at much lower surface area dose levels compared to insoluble NMs 1 day post-exposure.

**Fig. 5:**
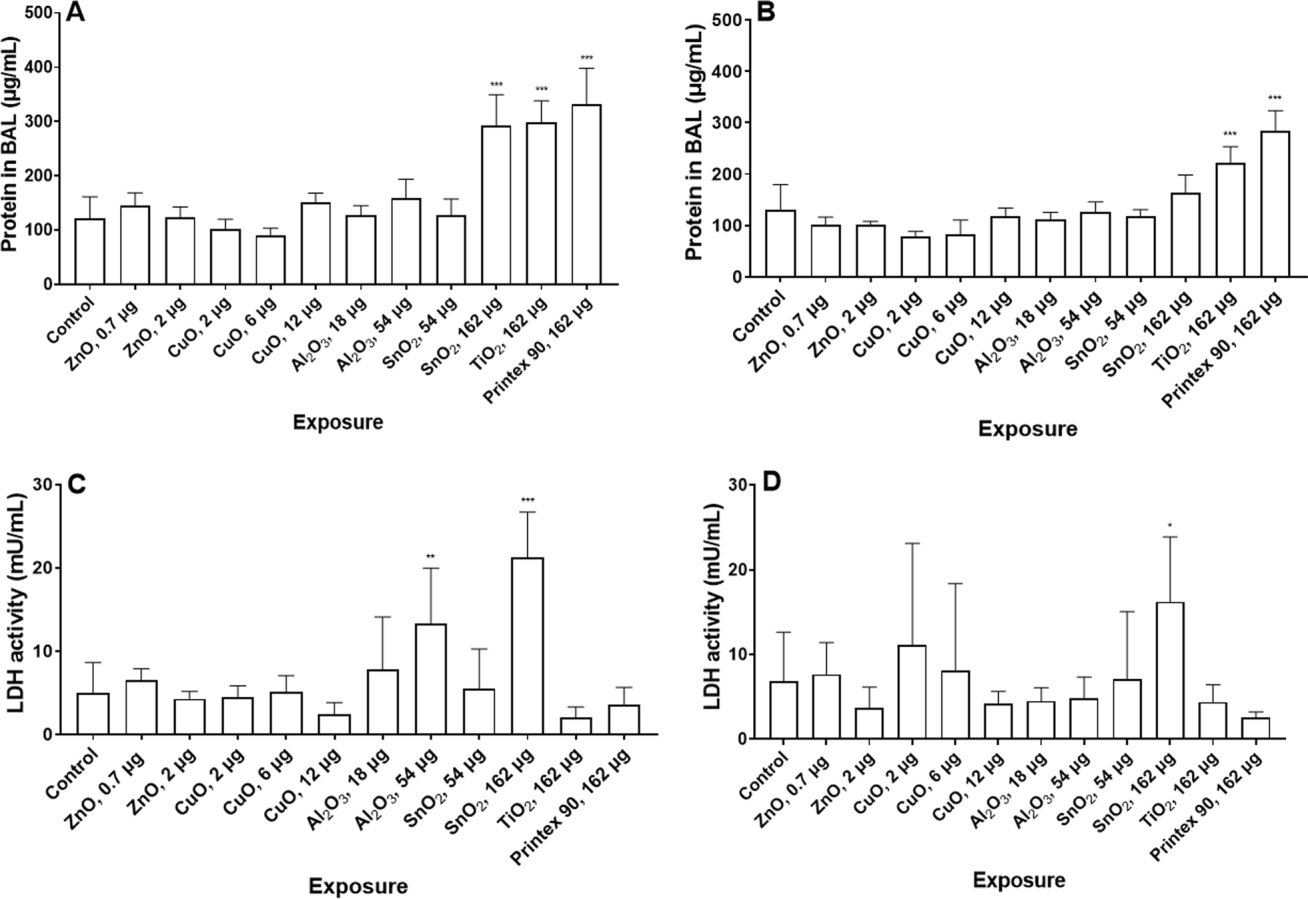
Total protein content in BAL fluid 1 (**A**) and 28 (**B**) after exposure to NM. LDH level in BAL fluid 1 (**C**) and 28 (**D**) after exposure. Data are shown as mean and bars represent SD. *, ** and *** designate *p *values < 0.05, < 0.01 and < 0.001 respectively vs. control

We measured total protein content and LDH activity in BALF to assess tissue injury. Figure 5 shows that after 1 day of exposure mice exposed to the high dose level of SnO_2_, TiO_2_, and Printex 90 presented a statistically significant increase in protein level in BALF compared to the control group. After 28 days, only the groups of mice exposed to TiO_2_ and Printex 90 presented a statistically significantly increased level of protein in BALF. We obtained significant correlations between neutrophil numbers and protein content in BALF, 1 and 28 days post exposure (day 1: r = 0.85, *p *value < 0.001; day 28: r = 0.81, *p *value: 0.001). Total protein content presented also a strong correlation with dosed surface area (day 1: r = 0.94, *p *value < 0.001; day 28: r = 0.94, *p *value: 0.001).

In the case of LDH activity in BALF (Fig. 5C and D), a significantly increased LDH activity was observed in mice 1 day after exposure to the high dose levels of Al_2_O_3_ and SnO_2_. After 28 days, only the group of mice exposed to the high dose level of SnO_2_ presented a statistically significant high activity of LDH in BALF compared to the control group. Taken together, these results indicate the presence of tissue injury after exposure to SnO_2_, TiO_2_ and Printex 90, however cytotoxicity was induced by Al_2_O_3_ and SnO_2_.

### *Saa3* and *Saa1* mRNA levels

**Fig. 6:**
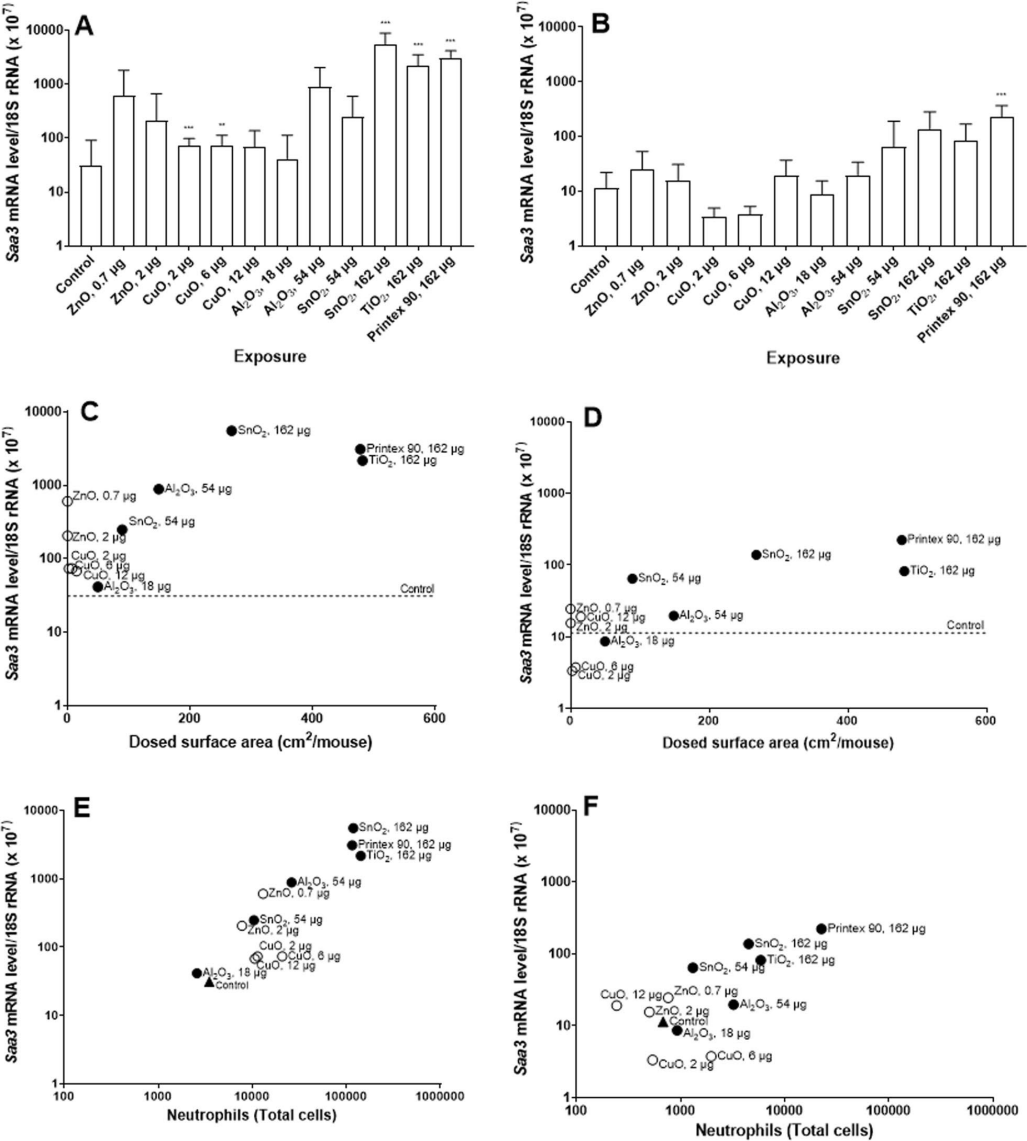
*Saa3* mRNA levels in lungs, 1 (**A**) and 28 (**B**) days after exposure to NM. Data are shown as mean and bars represent SD. ** and *** designate *p *values < 0.01 and < 0.001 respectively vs. control. Correlations between dosed surface area and *Saa3* mRNA levels, 1 (**C**) and 28 (**D**) days after exposure. Correlations between neutrophil numbers and *Saa3* mRNA levels, 1 (**E**) and 28 (**F**) days after exposure to NM

To investigate the induction of pulmonary and hepatic APR, we measured mRNA levels of *Saa3* and *Saa1*, respectively. Figure 6A and B show *Saa3* mRNA levels in lung tissue after exposure to NM. After 1 day, we observed a statistically significant increase of *Saa3* expression levels in mice exposed to low and medium CuO dose levels, high dose level of SnO_2_, TiO_2_ and Printex 90. However, Printex 90 was the only NM that induced a significant and persistent increase in *Saa3* mRNA levels 28 days post-exposure.

*Saa3* mRNA levels in lung tissue were correlated to NM surface area (Fig. 6C and D). We found a statistically significant correlation between the *Saa3* mRNA levels and pulmonary deposited particle surface area expressed as dosed particle surface area per mouse for both time points after exposures (day 1: r = 0.88, *p *value < 0.001; day 28: r = 0.83, *p *value < 0.001). When subdivided by solubility, the soluble NMs increased *Saa3* mRNA levels at lower surface area dose levels than the insoluble NMs 1 day post-exposure. Additionally, *Saa3* mRNA levels correlated strongly with neutrophil numbers across NMs (Fig. 6E and F), doses and time points (day 1: r = 0.95, *p *value < 0.001; day 28: r = 0.76, *p *value: 0.004).

**Fig. 7:**
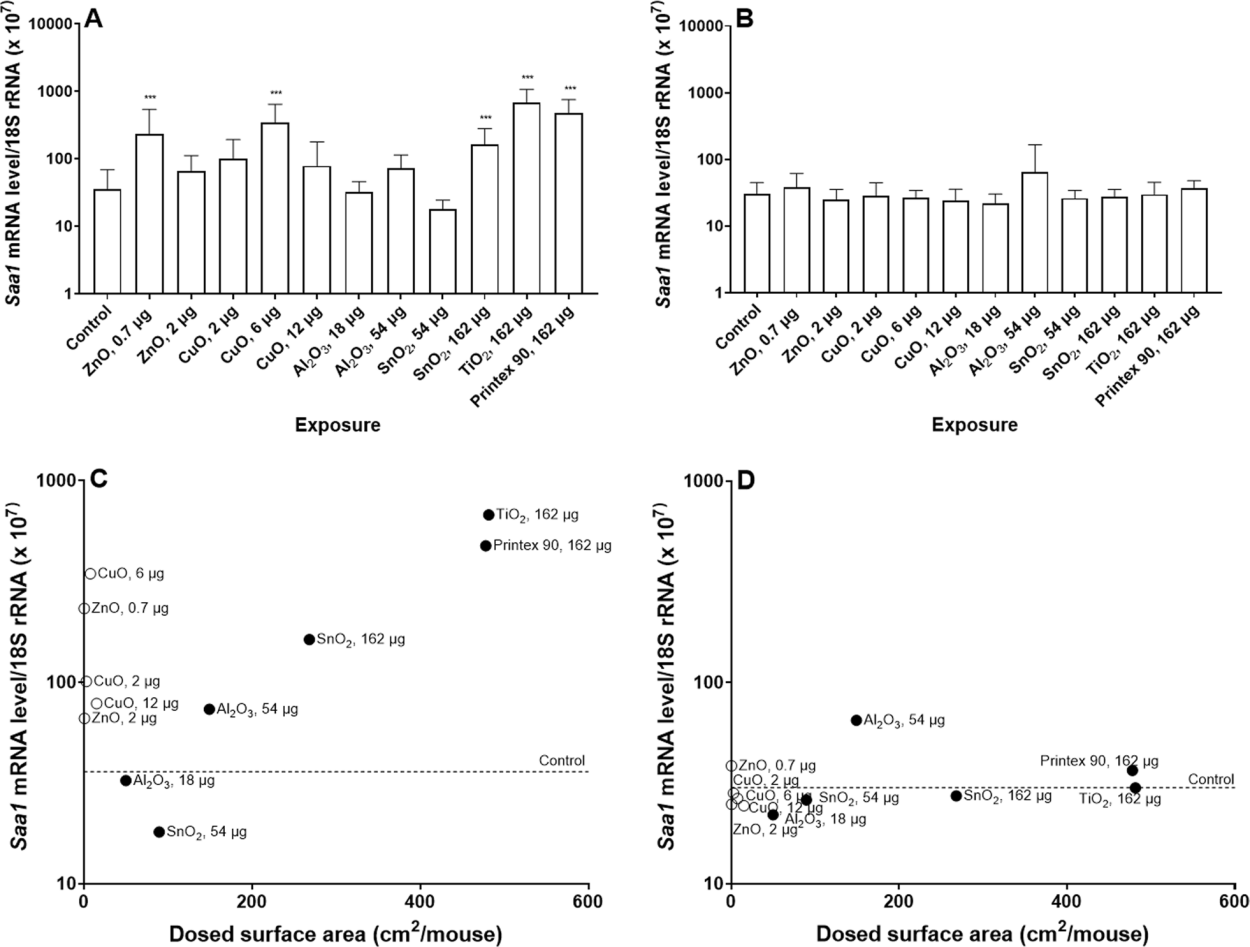
*Saa1* mRNA levels in liver, 1 (**A**) and 28 (**B**) after exposure to NM. Data are shown as mean and bars represent SD. ** and *** designate *p *values < 0.01 and < 0.001 respectively vs. control. Correlations between dosed surface area and *Saa1* mRNA levels, 1 (**C**) and 28 (**D**) days after exposure

In the case of hepatic APR (Fig. 7), *Saa1* mRNA levels in liver tissue were statistically significantly increased in the group of mice exposed to the low dose level of ZnO, medium dose level of CuO, high dose level of SnO_2_, TiO_2_ and Printex 90 one day post-exposure. After 28 days, there was no significant difference between the control group and NM exposed groups. *Saa1* mRNA level correlated with dosed NM surface area only at day 1 (r = 0.7, *p *value: 0.012).

Taken together, these results suggest that exposure to metal oxide NMs induced dose-dependent APR in lung and liver in mice. At 1 day post-exposure, highly soluble NMs seemed to induce stronger pulmonary and hepatic APR by dosed surface area than insoluble NM. None of the studied metal oxide particles induced APR 28 days post-exposure.

### SAA3 and SAA1/2 protein levels

**Fig. 8:**
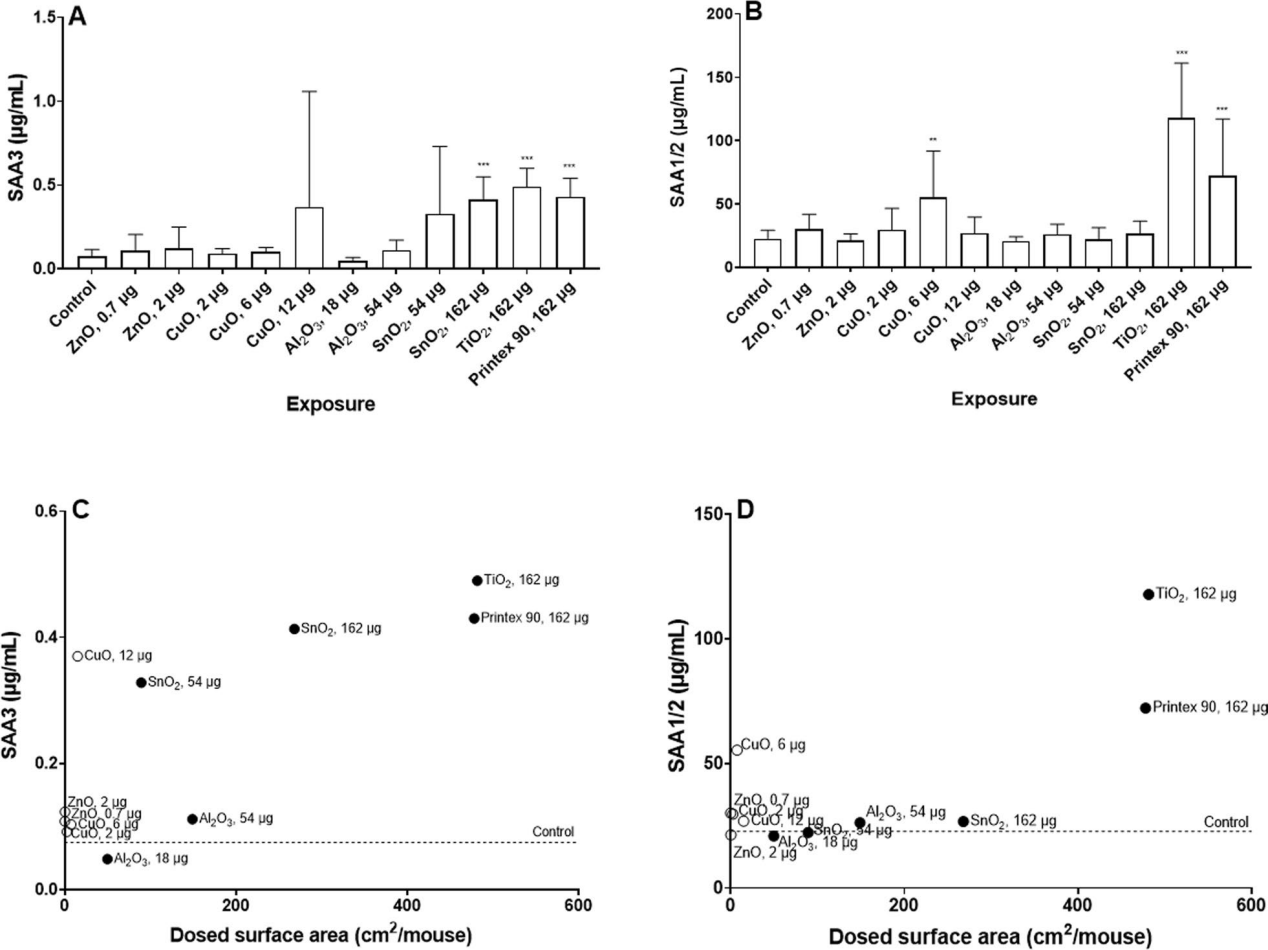
SAA3 (**A**) and SAA1/2 (**B**) protein levels in plasma, 1 day after exposure to NM. Data are shown as mean and bars represent SD. ** and *** designate *p *values < 0.01 and < 0.001 respectively vs. control. Correlations between dosed surface area and SAA3 (**C**) and SAA1/2 (**D**)

For investigating systemic APR induction, we measured plasma levels of SAA3 and SAA1/2. We found that plasma levels of SAA3 (Fig. 8A) were statistically significantly increased in mice exposed to the high dose level of SnO_2_, in addition to the mice exposed to TiO_2_ and Printex 90. In the case of SAA1/2 (Fig. 8B), exposure to a medium dose level of CuO, and exposure to TiO_2_ and Printex 90 showed a statistically significantly increased level of SAA1/2 in plasma.

When evaluating SAA protein level results in relation to *Saa3* and *Saa1*, we observed that SAA3 levels in plasma were significantly correlated to *Saa3* mRNA levels in lung tissue (r = 0.92, *p *value < 0.001), while SAA1/2 levels in plasma were significantly correlated to *Saa1* mRNA level in liver tissue (r = 0.91, *p *value < 0.001). When the proteins were evaluated against the opposite mRNA level, we observed less correlation but it was still statistically significant (*Saa3* and SAA1/2: r = 0.61, *p *value: 0.03; *Saa1* and SAA3: r = 0.66, *p *value: 0.02).

In addition, we evaluated the correlation of SAA3 and SAA1/2 plasma level in relation to dosed NM surface area, and found a statistically significant correlation in both cases (SAA3: r = 0.88, *p *value < 0.001; SAA1/2: r = 0.77, *p *value: 0.003), as shown in Fig. 8C, D.

### Correlations with surface area and dose

In addition to the results obtained in this study, we collected results from ten previous studies, where mice were exposed by a single intratracheal instillation to a vehicle suspension, metal oxide NMs or carbon black (Printex 90).

The same protocol for intratracheal instillation was used in all studies. The metal oxide NMs include ZnO, CuO, Fe_2_O_3_, Fe_3_O_4_, Co_3_O_4_ and rutile and anatase TiO_2_ [14, 17, 30–33, 36–38, 47, 53]. In all the studies, female C57BL/6J mice of approximately the same age (8 to 9 weeks old) were followed for 1 or 28 days and neutrophil numbers, pulmonary *Saa3* mRNA levels, hepatic *Saa1* mRNA levels and/or SAA3 plasma protein levels were assessed. Only the present study assssed SAA1/2 plasma protein levels. The information of the studies are presented in Additional file 9: Table S6. The metal oxides were subdivided according to solubility, i.e. ZnO and CuO as soluble and the rest of NMs (i.e. Al_2_O_3_, SnO_2_, Fe_2_O_3_, Fe_3_O_4_, Co_3_O_4_, TiO_2_ and Printex 90) as insoluble. We correlated the results obtained to the dose expressed as surface area or mass of each NM (Table 3). In addition, the *Saa3* mRNA and SAA3 protein levels in lung tissue and plasma from Jacobsen and colleagues (2015) were assessed and are presented in Additional file 10.

**Table 3:** Pearson correlation coefficient (r) obtained between outcomes and NM dose expressed as surface area or mass

Day	Outcomes	Log dosed surface area		Log dosed mass	
		Soluble NMs	Insoluble NMs	Soluble NMs	Insoluble NMs
Day 1	Log Neutrophils	0.61*	0.75***	0.47	0.73***
	Log *Saa3* mRNA level	0.25	0.70***	0.38	0.82***
	Log SAA3	0.50	0.92*	0.67	0.98***
	Log *Saa1* mRNA level	0.17	-0.04	0.23	0.27
	Log SAA1	0.34	0.83*	0.25	0.67
Day 28	Log Neutrophils	-0.64*	0.73***	-0.62*	0.56***
	Log *Saa3* mRNA level	-0.64*	0.32	-0.53	0.51**
	Log *Saa1* mRNA level	-0.68	0.66	-0.74	0.58

*p value < 0.05, **p value < 0.01, ***p value < 0.001

**Fig. 9:**
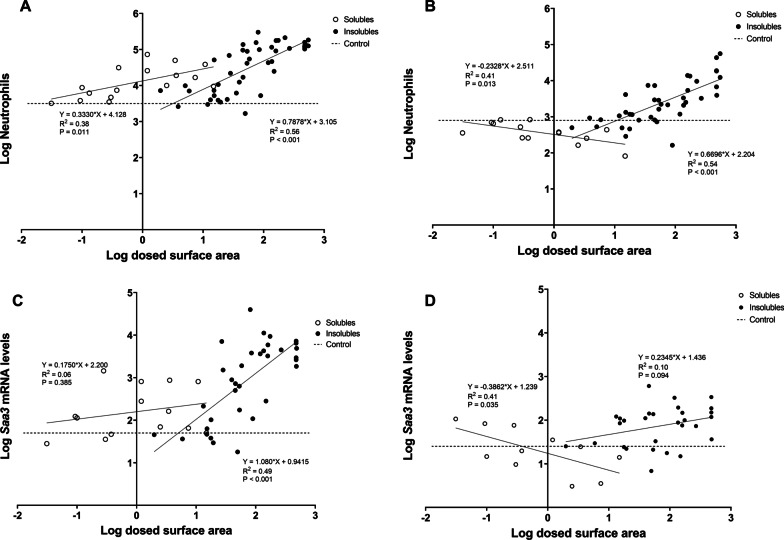
Correlations between dosed surface area and neutrophil numbers, 1 (**A**) and 28 (**B**) days after exposure, and dosed surface area and *Saa3* mRNA levels in lung tissue, 1 (**C**) and 28 (**D**) days after exposure. Results from the present study were combined with previously published data [14, 17, 30–34, 36, 37, 45, 53, 96]

When analyzing dose expressed as surface area, we obtained significant correlations when evaluating each group against neutrophil numbers, both at day 1 (*p *value: 0.011 for soluble NMs and *p *value < 0.001 for insoluble NM) and day 28 (*p *value: 0.013 for soluble NMs and p < 0.001 for insoluble NMs) (Fig. 9A and B). The soluble NMs induced neutrophil influx at low surface area dose levels as compared to insoluble NMs at day 1. However, at day 28, neutrophil influx was negatively correlated to dosed surface area of soluble NMs. When comparing with dose expressed as mass, insoluble NMs were significantly correlated with neutrophils at day 1 (*p *value < 0.001), while for both soluble and insoluble NMs, significant correlations were found for neutrophils at day 28 (*p *value: 0.019 for soluble NMs and *p *value < 0.001 for insoluble NMs) (see Additional file 11), although for soluble NMs an inverse correlation was found.

**Fig. 10:**
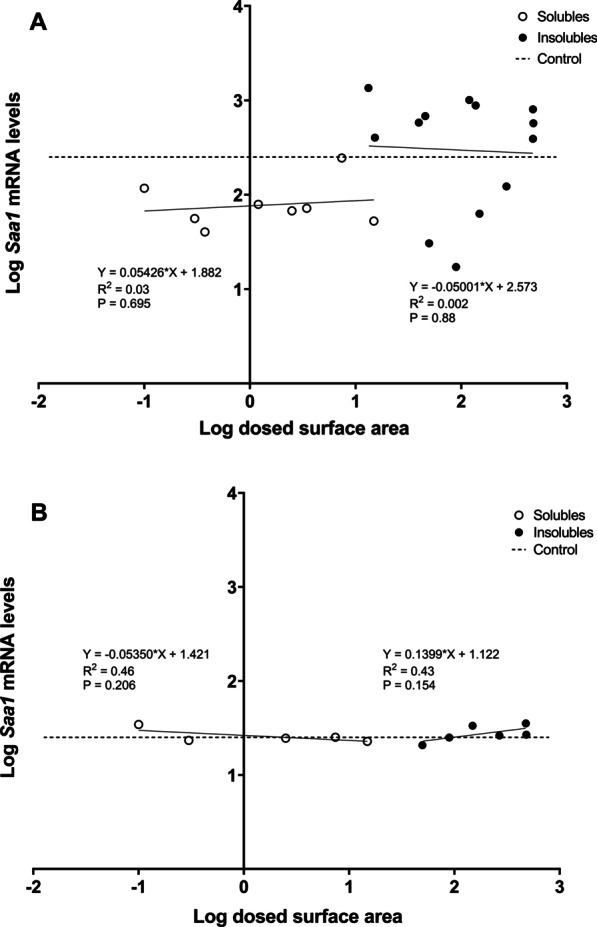
Correlations between between dosed surface area and *Saa1* mRNA levels in liver tissue, 1 (**A**) and 28 (**B**) days after exposure. Results from the present study were combined with previously published data [17, 30]

**Fig. 11:**
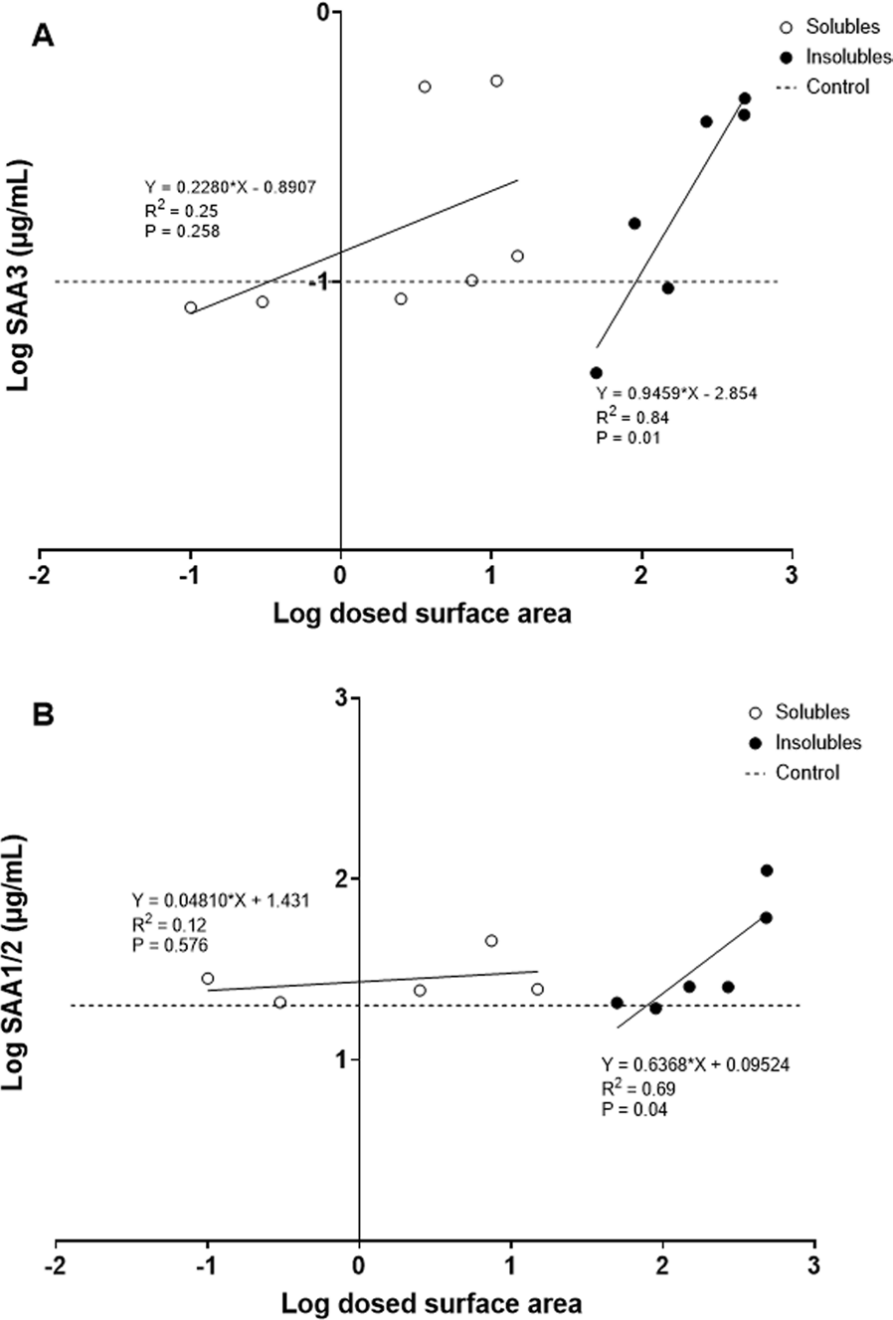
Correlations between pulmonary dosed surface area and SAA3 (**A**) and SAA1/2 (**B**) protein in plasma, 1 day after exposure. Results from the present study were combined with previously published data [37]

In case of *Saa3* mRNA levels at day 1 (Fig. 9C), only insoluble NMs were significantly correlated with dosed surface area (*p *value < 0.001); while for day 28 (Fig. 9D), *Saa3* mRNA levels were significantly but negatively correlated to the surface area of soluble NMs (*p *value: 0.035). Insoluble NMs dosed mass correlated with *Saa3* mRNA levels at days 1 and 28 (*p *value < 0.001 for day 1 and *p *value: 0.005 for day 28), while soluble NMs dosed mass had no correlation with *Saa3* mRNA levels at days 1 nor 28. *Saa1* mRNA levels in liver were not correlated with dosed particle surface area regardless of the NMs solubility, and surface area explained very little of the observed variation as r values were very low (Fig. 10). Similarly, we did not observe any significant correlations between NMs dosed in mass and *Saa1* mRNA levels. In the case of SAA3 and SAA1/2 plasma protein levels (Fig. 11), only insoluble NMs had significant correlations with dosed surface area (*p *value: 0.001 for SAA3 and *p *value: 0.04 for SAA1/2). In the case of dosed mass, SAA3 plasma protein levels correlated only with insoluble NMs (*p *value < 0.001), while no correlation with SAA1/2 plasma protein levels was seen neither for soluble nor insoluble NMs.

**Fig. 12:**
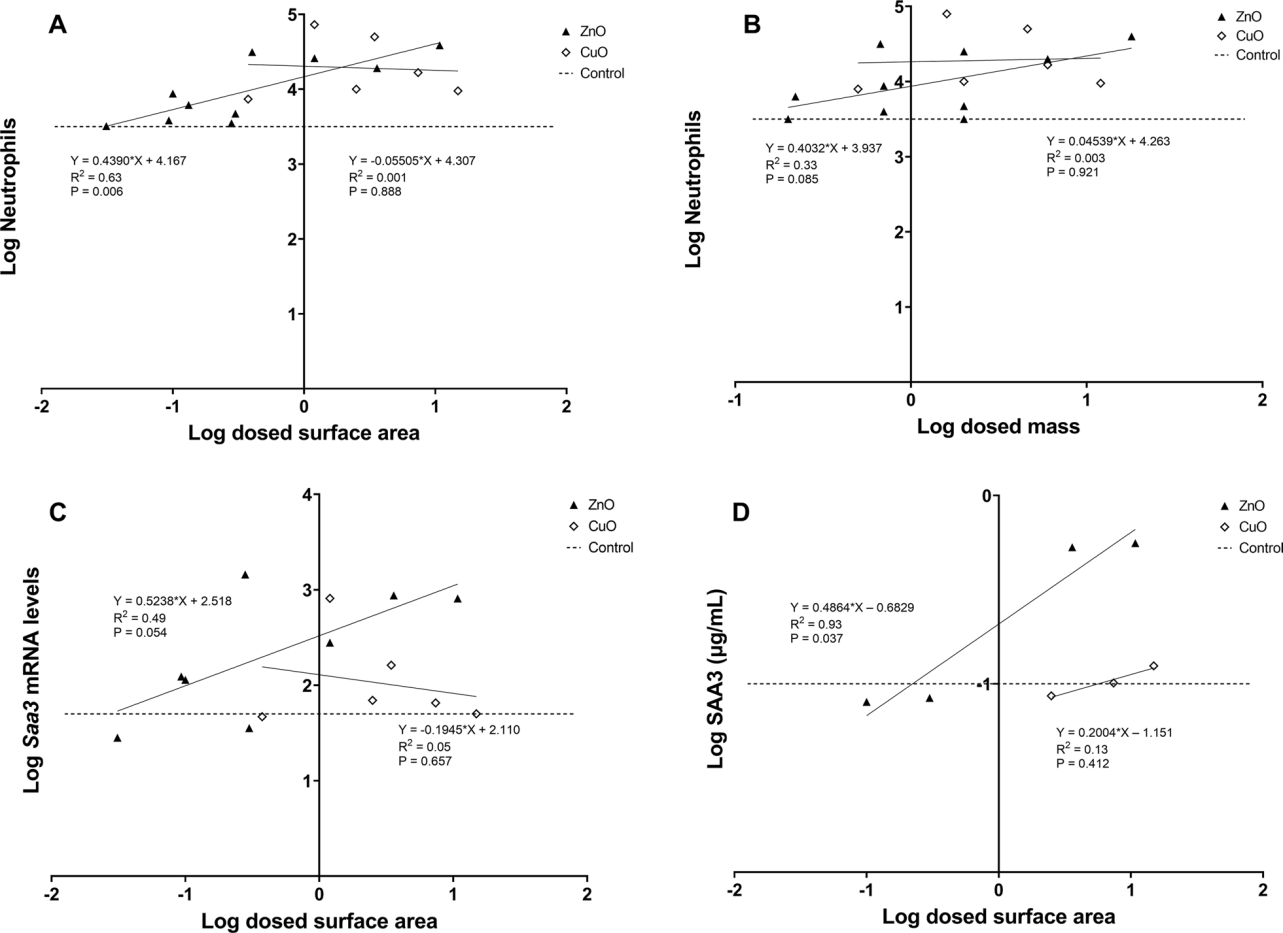
Correlations between ZnO and CuO: (**A**) dosed surface area and neutrophil numbers, (**B**) dosed mass and neutrophil numbers, (**C**) dosed surface area and *Saa3* mRNA levels in lung tissue, and (**D**) dosed surface area and SAA3 plasma protein levels. All data considers 1 day after exposure to either ZnO or CuO. Results from the present study were combined with previously published data [14, 30, 37]

ZnO and CuO have different solubility kinetics (Table 2), and therefore, ZnO and CuO were also evaluated separately (see Fig. 12 and Additional file 11). For CuO NMs, there was no significant correlation between dosed particle surface area and the different endpoints, while ZnO NMs showed significant correlations between dosed particle surface area and neutrophil numbers at day 1 (*p *value: 0.006) and SAA3 plasma protein levels (*p *value: 0.037). We could not analyze *Saa1* mRNA levels nor SAA1/2 plasma protein levels due to small data sets for ZnO NMs. When evaluating ZnO and CuO dosed as mass, only one significant correlation was found between ZnO and *Saa3* mRNA levels (*p *value: 0.044).

**Fig. 13:**
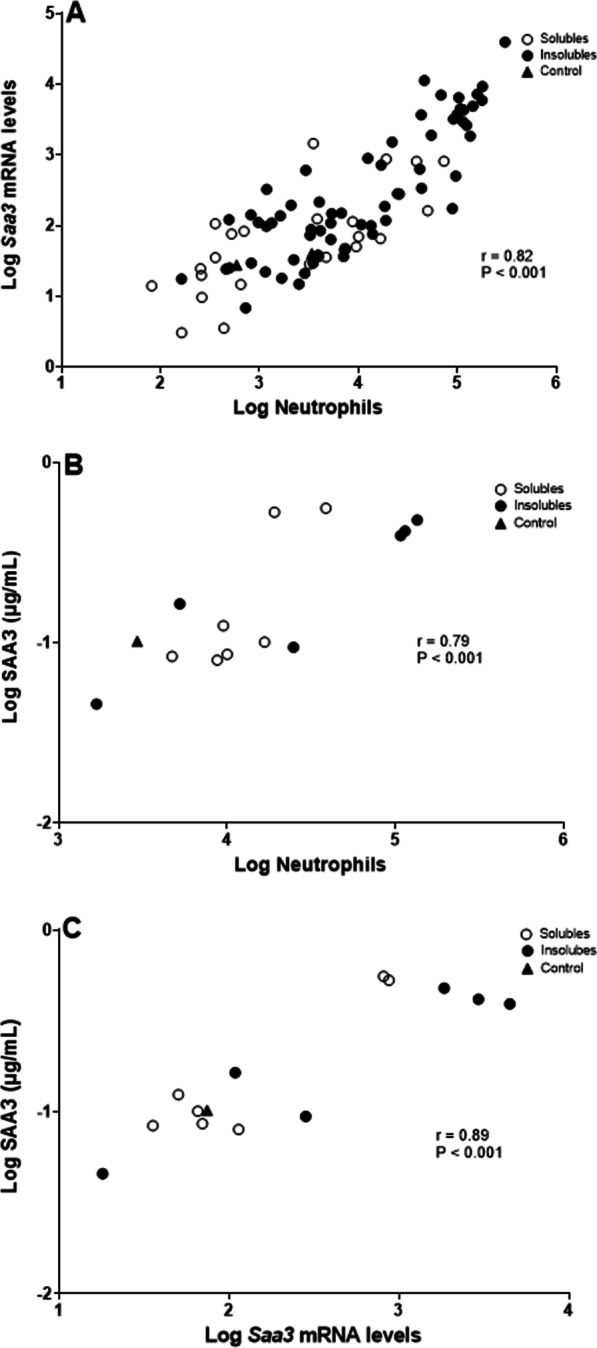
Correlations between: (**A**) Neutrophil numbers and *Saa3* mRNA levels in lung tissue, including data from 1 and 28 days after exposure to NM; (**B**) Neutrophil numbers and SAA3 plasma protein levels, including data from 1 day after exposure to NM; and (**C**) *Saa3* mRNA levels in lung tissue and SAA3 plasma protein levels, including data from 1 day after exposure to NM. Results from the present study were combined with previously published data [14, 17, 30–34, 36, 37, 45, 53, 96]

Finally, when combining all data from day 1 and day 28 for all NMs, we obtained a strong, significant correlation between neutrophil numbers and *Saa3* mRNA levels (r = 0.82, *p *value < 0.001) (Fig. 13A). In the case of correlations with SAA3, where we only have data from day 1, our results also showed significant correlations between neutrophil numbers and SAA3 plasma protein levels (r = 0.79, *p *value < 0.001) (Fig. 13B), and *Saa3* mRNA levels and SAA3 plasma protein levels (r = 0.89, *p *value < 0.001) (Fig. 13C); indicating that these parameters can be used as interchangeable biomarkers for APR after exposure to soluble and insoluble particles.

## Discussion

SAA is one of the most differentially regulated proteins during APR and an established risk factor for CVD [8, 11, 54]; since it promotes plaque progression, we have proposed that it may constitute a causal link between inhalation of particles and CVD risk [11–13]. In the present study, mice were exposed by intratracheal instillation to five different metal oxide NMs (ZnO, CuO, Al_2_O_3_, SnO_2_ and TiO_2_) and we analyzed inflammation and APR induction, 1 or 28 days after exposure. We have reported pulmonary and hepatic acute phase responses using *Saa3* and *Saa1* mRNA levels a biomarkers of the pulmonary and hepatic acute phase responses, respectively, as well as increased SAA plasma levels following exposure to soluble and insoluble NMs. Furthermore, we show that SAA3 protein levels correlated with *Saa3* mRNA levels and neutrophil numbers. Both soluble and insoluble metal oxides induced dose-dependent APR in lungs, but their time-dependence differed. While soluble metal oxides induced a stronger pulmonary APR than insoluble metal oxides at 1 day post-exposure, the pulmonary APR induced by insoluble metal oxides was more long lasting, as increased *Saa3* mRNA levels were observed 28 days post-exposure, as also previously reported for TiO_2_ NMs [17, 33]. The long-lasting increase in *Saa3* and SAA3 has previously been reported for other insoluble NMs such as MWCNT [16, 55]. These results suggest that both soluble and insoluble metal oxide NMs induce pulmonary APR, but with different time-dependency. Thus, we demonstrated that all the studied metal oxide NMs induce APR, thus providing evidence that links exposure to metal oxides to risk of CVD [12].

In the case of ZnO and CuO NM, we observed that only the lowest dose level of ZnO (0.7 µg) induced neutrophil influx. Similarly, this dose increased *Saa1* mRNA levels, while none of the ZnO doses increased *Saa3* nor increased plasma levels of SAA3 or SAA1/2. We also found that pulmonary exposure to CuO NMs induced lung inflammation 24 h after exposure, along with upregulating APR genes in lungs and liver and increasing SAA1/2 protein in plasma.

In a previous study, where the same doses of uncoated ZnO were used, an increase in neutrophil numbers was not observed, however there was an increase in *Saa3* mRNA levels in lung tissue at the highest dose level (2 µg) 1 day after exposure, which did not translate to an increase in plasma SAA3 levels [14]. As part of the present study, we analyzed *Saa3* mRNA in lung tissue and SAA3 plasma protein levels from a previous investigation, where mice were exposed to higher concentrations of ZnO (i.e. 2, 6 and 18 µg/mouse) (more details can be found in Jacobsen et al. [37]). At these higher dose levels, increased *Saa3* mRNA levels and SAA3 plasma protein levels were found 1 day after exposure (see Additional file 10).

It has also been shown that neutrophil influx in mice and rats is higher 3 days after intratracheal instillation of ZnO NM, as compared to 1 day post-exposure. In case of rats, neutrophil counts were observed to return to almost control levels one week after instillation [37, 56]. We also showed that exposure to ZnO increased *Saa1* mRNA levels in liver as biomarker for a hepatic APR [11, 16].

In the case of CuO, our results are in accordance with our previous study, where mice exposed to another CuO NM had increased neutrophil numbers and *Saa3* mRNA levels in lungs after 24 h, even at lower dose levels (i.e. 1.6 and 4.7 µg/mouse). In that study, *Saa3* mRNA remained increased after 28 days [30]. Lung inflammation has also been shown to occur in rats after CuO NM instillation in addition to increased LDH and total protein level in BALF 24 h after exposure, and elevated concentrations of IL-1β, MIP-2 and MCP-1 [57–59]. Finally, in an inhalation study performed by Gosens and colleagues [60], a dose–response relationship on lung inflammation was observed 1 day after rats were exposed to CuO NM for 5 days. Similar to our study, the inflammation also resolved during the recovery time of 22 days. A following transcriptomics study found that inflammation was one of the main processes occurring in the lungs of exposed rats, highlighted by the upregulation of both *ccl2* gene and CCL2 protein [61].

In the case of Al_2_O_3_, we have seen that even though the highest dose level triggers pulmonary inflammation, this NM did not induce pulmonary, hepatic nor systemic APR in mice. However, Al_2_O_3_ NM seems to induce cytotoxicity in the lungs. In a previous study with mice it was shown that exposure to Al_2_O_3_ increased LDH level from BALF 13 weeks after exposure, along with IL-6, MIP-1a and GM-CSF; but a high number of neutrophils was not observed [62]. In another study, Lu et al. [63] tested 4 different Al_2_O_3_ NMs in rats, and found that only 1 type of Al_2_O_3_ induced inflammation 24 h after intratracheal instillation, with no changes in LDH activity in BALF. It is important to note that although Al_2_O_3_ did not exhibit the highest response in the present study_,_ Sikkeland et al. [64] showed that human exposure to Al_2_O_3_ increased the number of neutrophils and IL-6 in induced sputum. Finally, experiments where rats have been repeatedly exposed to Al_2_O_3_ have showed that a single exposure was not sufficient to induce an effect on lung toxicity or inflammation, while repetitive exposure produced inflammation and increased LDH and pro-inflammatory cytokine levels in BALF. High levels of neutrophils were still observed after a 28 days recovery period [65, 66]. In this study, Al_2_O_3_ has been grouped with the insoluble NMs as it induced pulmonary inflammation 28 days after exposure, although having an intermediate solubility in PSF.

Stannosis is a benign form of pneumoconiosis produced by the inhalation and accumulation of tin in the lungs [67, 68]. To our knowledge, our study is the first one where animals have been exposed to SnO_2_ NM. We observed that this NM produces inflammation and induces pulmonary, hepatic and systemic APR in mice, as shown by the increased levels of neutrophils and plasma SAA3, almost as high as our benchmark materials (TiO_2_ and Printex 90). In vitro studies with SnO_2_ NM have shown that this material decreases cell viability, affects cell membrane structure and increases reactive oxygen species (ROS) production in different cancer cell lines, including a human lung adenorcarcinoma cell line (A549) [69–71]. A recent study compared the effects of SnO_2_ and TiO_2_ NM on A549 cell and murine monocytes. Despite their similar chemical characteristics, the researchers observed a higher uptake of TiO_2_ NM by both cell lines in comparison to SnO_2_; however, only a slight decrease in cell viability on monocytes exposed to TiO_2_ was seen [72].

We observed that our benchmark NMs (i.e. TiO_2_ and Printex 90) induced the highest responses in most of our end-points, including neutrophil influx, protein content in BALF, upregulation of *Saa3* and *Saa1* and SAA3 and SAA1/2 protein levels. Additionally, elevated markers of pulmonary APR was observed 28 days after pulmonary exposure to Printex 90. These results go along with several of our previous studies, where we have found that these NMs induce inflammation and APR in mice [14, 16, 17, 30, 33, 36, 48, 53].

No histopathological changes were observed in lungs of animals exposed to soluble NMs, while in the case of insoluble NMs macrophage aggregates and lymphocytic infiltrations were present. It is interesting to note that we did not find evidence of presence of Al_2_O_3_ particles in lung tissue 28 days post-exposure. Al_2_O_3_ has relatively low solubility in PSF, but the present data suggest that even limited solubility may contribute to the removal of particles from the lung. In the liver, inflammatory cell infiltrations are a common finding in mice, but exposure to chemicals can influence their incidence and severity [73]. The elevated numbers of inflammatory cell infiltrations 28 days post-exposure to CuO or SnO_2_ could be related to APR evoked by these NMs. We have recently reported that for carbon-based engineered NMs, liver transcription of the APR *Saa1* and *Lnc2* were dependent on toll-like receptor 2 (TLR-2) [74]. TLR-2 signaling also plays a critical protective role against acute *Listeria monocytogenes* (Lm) infection by up-regulating inflammatory cytokines and promoting macrophage antimicrobial capabilities [75]. It has been shown that Kupffer cell activation and macrophage infiltration in response to Listeria infection is mediated by TLR-2 dependent secretion of *Cxcl1* and *Ccl2* and that the macrophage infiltration is also stimulated by exogenous *Ccl2* and *Cxcl1* capabilities [75]. The Kupffer cells are activated by direct interaction with bacteria or LPS and therefore possibly also by translocating ENMs [74]. However, it is also possible that the combination of differences in particle translocation rates combined with differences in blood levels of CCL2 and CXCL1 caused by differences in the pulmonary inflammatory response could contribute to the differences in occurrence of inflammatory infiltrates in the liver. We have previously reported that pulmonary exposure to carbon back and titanium dioxide both increased protein levels of CXCL1 and CCL2 in lung tissue of exposed mice [20, 21] and demonstrated that a number of pulmonary exposure to a number of engineered NMs increases pulmonary transcription levels of *Ccl2* and *Cxc1* [20, 21]. This would predict that the high number of inflammatory infiltrates in the liver observed following exposure to the SnO_2_ NMs would be accompanied by significant particle translocation (having the smallest primary size of 4.5 nm) and possibly a pulmonary inflammatory response characterized by high expression of *Ccl2* and *Cxcl1*. The lack of enhancing effect on inflammatory cell infiltrations on day 28 post-exposure by TiO_2_ or Printex 90 is in accordance with our previous results [76]. No effect on inflammatory cell infiltrations in the liver of mice after exposure to Al_2_O_3_ is in accordance with our present finding of lack of any effect on local or systemic APR markers in this study, and may be due to the intermediate solubility of Al_2_O_3_.

We supplemented our results on inflammation and APR with results from ten previous studies, and correlated these to dose expressed as surface area or mass. We observed that inflammation caused by both soluble and insoluble NMs (i.e. neutrophil numbers) correlated with dosed surface area and mass, as our previous studies have shown [15, 16, 48]. However, there was very little difference in the particle sizes of the studied particles, so the dataset did not allow us to distinguish between mass or surface area as predictors of APR. Regardless, the present results corroborated previous studies showing that surface area can be used as a predictor of inflammation [77–80] and APR induction [11, 13] after pulmonary exposure to insoluble NM. While the soluble NMs gave a stronger response, the dose–response relationship may depend on the chemical composition of the soluble particles as observed for ZnO and CuO. On the other hand, the insoluble NMs seems to give more consistent inflammatory and APR effects with less strong dose–response relationship, but being more persistent. This is likely caused by the longer retention time in the lungs and has retained surface area as predictor of inflammatory and APR. In addition, we found that neutrophil numbers can be used as biomarkers for pulmonary *Saa3* mRNA and plasma SAA3 levels after pulmonary particle exposure, as all three were highly inter-correlated. This allows comparison of data from controlled human studies [23, 24, 81–84] where blood levels of SAA and/or CRP are available with data from rodent inhalation studies, where data on neutrophil influx are typically available.

The present study presents some limitations: 1) Although all the studies we used for the correlations were performed using the same mice strain, sex and age, and followed the same protocols in the same facilities, we cannot disregard the small variation each experiment might have had as a limitation. Although different dispersion media were used, we have previously assessed the effects of different vehicles on TiO_2_, carbon black NM and on CNT induced lung toxicity, and have found that the vehicle has a minor effect on the particle-induced inflammatory response [39]. Accordingly, neutrophils numbers from mice exposed to these vehicle controls or our benchmark material (Printex 90), showed similar results 1 day after exposure, regardless of the dispersion medium used (Additional file 11: Figure S9). 2) In the case of the soluble NMs, high dose levels were not used for animal ethical reasons, while for the insoluble NMs low dose level were not included. The lower dose levels for ZnO and CuO NMs came from previous studies, where we observed acute toxicity following pulmonary dosing of ZnO and CuO [30, 37]. On the other hand, we used the higher dose levels for the benchmark materials (i.e. TiO_2_ and Printex 90) because previous studies have shown that using lower doses levels have no or little effect on pulmonary inflammation and APR and they are not long lasting [34, 43]. 3) Some of the metal oxides NMs exposures did not produced a significantly significant effect on the end-points evaluated. However, we decided to combine all data to assess if two factors (i.e. mass/surface area and inflammation/acute phase response) co-vary. In addition, we also kept data points that were at the same level as vehicle controls as this can be used to identify no-effect levels and provide evidence of presence or absence of threshold effects.

In 2020, Hadrup and colleagues presented a list of biomonitoring studies which related the exposure to particles to SAA and CRP. The list included diverse exposures as ZnO, welding fumes, organic dust emissions from paper, iron and steel production, coke-ovens, firefighting and diesel engine exhaust. In addition to the studies presented in that list, other studies need to be mentioned; Walker and colleagues (2020) assessed the effects of PM_2.5_ emissions from different types of stoves types in volunteers. They observed a dose-dependent correlation between wood stove emissions and blood levels of CRP and SAA level, in volunteers 24 h after exposure, where 2 h of exposure to 0.5 mg/m^3^ wood stove emissions increased CRP and SAA levels in blood, whereas exposure to 0.25 mg/m^3^ and lower exposure levels did not. In turn, Wyatt and colleagues (2020) exposed volunteers to 0.038 mg/m^3^ ambient PM_2.5_, obtaining increases in SAA and CRP, 1 h after exposure; 20 h after exposure SAA remained elevated, while Andersen et al. showed that exposure to 0.0085 mg/m^3^ diesel exhaust measured as elemental carbon did not increase CRP or SAA levels [85, 86]. Taken together, this evidence suggests that exposure to particles leads to induction of APR in human volunteers.

Monsé and colleagues recently published results from a controlled exposure study, where human volunteers were exposed to microsized or nanosized ZnO for 2 h on separate exposure days [81]. 24 h after onset of exposure, CRP levels were increased from 0.77 mg/L to 1.87 or 2.23 mg/L for nano and micro-sized ZnO, respectively. The alveolar deposited dose was calculated to be 0.62 and 1 mg, respectively, while the specific surface areas were 20.2 and 4.8 m^2^/g, respectively, for the nano and microsized ZnO. This suggests that for the highly soluble ZnO particles, mass is a better predictor for APR than deposited surface area, as the almost three times higher dosed surface area for the nanosized ZnO induced less APR (0.00062 g × 20.2 m^2^/g = 0.013 m^2^ for nano and 0.001 g × 4.8 m^2^/g = 0.0048 m^2^ for the micrometer sized ZnO).

In a previous study [23], Monsé and colleagues exposed human volunteers to different concentrations of ZnO nanoparticles for 4 h, and 0.5 mg/m^3^ was identified as the No Observed Adverse Effect Level (NOAEL) for CRP blood level. The estimated deposited ZnO was 10 m^3^/8 h × 4 h × 0.5 mg/m^3^ ZnO × 0.48 (alveolar deposition rate) = 1.2 mg, corresponding to a dose of 1.2 mg/72 kg body weight (bw) = 0.017 mg/kg bw. The corresponding dose for total pulmonary deposited dose was 0.027 mg/kg bw (using the deposition rate for tracheobronchial, inhalable and alveolar deposition combined of 78%). In the present study, we observed a significant effect on neutrophils numbers at 0.7 µg/mouse but not at 2 µg/mouse, and if we use these dose levels as a range for the dose-dependent NOAEL for neutrophil numbers, we obtain a dose level of 0.035 to 0.1 mg/kg bw, while the NOAEL for *Saa3* mRNA levels would be 2 µg/mouse (0.1 mg/kg bw) and 2 µg/mouse for SAA3 in plasma (0.1 mg/kg bw). We consider these estimates as being in the same order of magnitude as the NOAEL from Monsé and colleagues. This, in turn, may suggest that data from mice may be used for risk assessment in relation to particle-induced APR. Based on the controlled exposure study by Monsé and colleagues [81], a health-based occupational exposure limit for ZnO of 0.04 mg/m^3^ was proposed by the National Research Centre for the Working Environment [87].

CRP and SAA are considered risk factors for CVD [1, 2]. While CRP is commonly measured in humans and is a major acute phase protein [1], CRP is only a minor acute phase protein in mice [88, 89]. We selected SAA as biomarker of APR, because *Saa3* was the most upregulated gene in the lungs both after inhalation and instillation exposure to particles and NMs, although *Saa1, Saa2* and *Saa3* all are among the most differentially regulated genes following pulmonary particle exposure [11, 13, 44, 90, 91]. In addition, studies have shown that genetic variation in the promoter region of CRP gene which predicts CRP levels, is not associated with CVD [92–94], suggesting that CRP co-varies with the causal factor in relation to CVD; as SAA highly correlates with CRP [95], SAA might be the causal factor CRP in question. The SAA family has been evolutionary conserved [3]. In humans, SAA1 and SAA2 are the two major isoforms [8]; murine SAA1 and SAA2 isoforms are highly homogenous to their human counterpart, while mouse SAA3. which is not expressed in humans, is also highly homogenous to mouse SAA1 and SAA2 isoforms [3]. Although SAA3 is not expressed in humans, our findings suggest we can use neutrophil numbers, *Saa3* mRNA level in lung tissue and SAA3 plasma protein as interchangeable biomarkers of particle-induced APR in animal models, due to their high correlations, as biomarkers of particle-induced APR for risk assessment of metal oxide NMs exposure in relation to CVD.

## Conclusions

In this study, we have shown that soluble and insoluble NMs induce pulmonary inflammation and APR. Particle solubility (or particle persistence) was identified as an important predictor of particle-induced APR. For soluble NMs, APR was induced at low dose levels and the APR was of short duration. For insoluble NMs, APR correlated with deposited surface area and was more long lasting (i.e. at least 28 days). In the case of ZnO, where controlled human studies are available, we found similar NOAEL values derived from human data and data from intratracheal instillation exposure in mice. In the present study, neutrophil influx, *Saa3* mRNA levels in lung tissue and plasma SAA3 levels correlated across all studied nanomaterials, suggesting that these endpoints can be used as interchangeable biomarkers of APR and CVD risk following exposure to soluble and insoluble particles.

## Supplementary Information

**Additional file 1.** Dissolution analysis for Al_2_O_3_ and SnO_2_.

**Additional file 2.** Figure S1. Particle equivalent circular diameter distributions from TEM measurements on five metal oxide samples.

** Additional file 3. Table S1**. Overview table of results from TEM analysis.

**Additional file 4. Figure S2**. Number-based hydrodynamic size distributon of NMs dispersed in instillation vehicle measured by dynamic light scattering.

**Additional file 5.** Figure S3. Mouse lung histopathology 28 days post-exposure to vehicle control, ZnO, CuO, Al_2_O_3_, SnO_2_, TiO_2_ and Printex 90.

**Additional file 6.** Table S2. Mean number and incidence of lymphocytic infiltrates and macrophage aggregates in lungs, 1 and 28 days post-exposure to NMs.

**Additional file 7.** Table S3 and Table S4. Type and incidence of histological changes in livers from mice 28 days after intratracheal exposure to vehicle control or NMs.

**Additional file 8.** Table S5. BAL cell count 1 and 28 days after exposure to NMs.

**Additional file 9.** Table S6. Publications used in the correlation analysis.

**Additional file 10.** Figures S4 and S5. *Saa3* mRNA levels in lung tissue and SAA3 protein levels in plasma from mice, 1 day after intratracheally instillation with 2, 6 or 18 µg of ZnO.

**Additional file 11.** Figures S6, S7, S8 and S9. Correlations between dosed mass and neutrophil numbers, *Saa3* mRNA levels, *Saa1* mRNA levels, SAA3 plasma protein levels or SAA1/2 plasma protein levels, and neutrophil number from vehicle control and Printex 90 from studies used in correlations.

## Data Availability

The datasets used and/or during the current study are available from the corresponding author on reasonable request.
