# Supplementary material for: Acute phase response following pulmonary exposure to soluble and insoluble metal oxide nanomaterials in mice
Source: Part Fibre Toxicol. 2023 Jan 17;20:4. doi: 10.1186/s12989-023-00514-0 (PMC9843849; doi:10.1186/s12989-023-00514-0)
Supplement: Supplementary file 1 — Additional file 1. Dissolution analysis for Al2O3 and SnO2. [file 12989_2023_514_MOESM1_ESM.docx]

Additional information 1

**Dissolution of test materials**

Methods

A*tmosphere-Temperature-pH-controlled Stirred Batch Reactor system*

Dissolution testings were performed using a ATempH SBR (Atmosphere-Temperature-pH-controlled Stirred Batch Reactor) system constructed with four separate and centrally controlled 120 mL double-walled glass reactors units. The system has been previously described in Holmfred et al. (2022). In testing, one reactor unit is used as a reference containing the pure test medium (Phagolysosomal Simulant Fluid, PSF) and the other three units are used for as three replicate dissolution testing of the NMs (n=3).

The test atmosphere (5,62 mL/min CO_2_ and 144 mL/min HEPA-filtered air) in the reactors were monitored and controlled via a MultiFlo Cable Kit mass-flow meter and controller (Brooks Instrument, Hatifield, PA, USA), respectively.

Each reactor unit has a separate OMNIS bi-directional titration module (Metrohm, Herisau, Switzerland) for continuous pH adjustment with 1 M HCl and 1 M NaOH (Reagecon Diagnostics Ltd., Country Clare, Ireland), respectively. The titration volume (recorded every 10 seconds) was used to calculate the acid/base dilution between each sampling time point during the 24 h dissolution study, depending on the NM and test medium).

The test atmosphere (5,62 mL/min CO_2_ and 144 mL/min HEPA-filtered air) in the reactors were monitored and controlled via a MultiFlo Cable Kit mass-flow meter and controller (Brooks Instrument, Hatifield, PA, USA), respectively.

The test temperature was held at 37 °C by using a PolyScience water bath pump system (Holm & Halby, Brøndby, Denmark) to circulate heated water continuously. To protect the NMs from light, each reactor was gently wrapped in aluminum foil.

pH-electrodes were used to measure pH during testing for regulation in the titration modules and Pt redox electrodes were used to measure redox potential data (Metrohm, Herisau, Switzerland). Before testing, the Pt redox electrodes were calibrated with a 250 mV solution, and the pH electrodes were calibrated with pH 4.0 and 7.0 solutions (Reagecon Diagnostics Ltd, Clare, Ireland).

The speed of the three-bladed propeller stirrer was set to 840 rpm throughout all experiments. Prior to testing, the PSF was adjusted to pH 4.5 , and kept constant throughout testing.

Testing was conducted by pre-dispersing the test materials using the so-called NANOGENOTOX dispersion protocol, which implies pre-wetting in 0.5 vol% Ethanol and dispersion by probe-sonication in 0.05 % wt/vol BSA water. See details below.

After the pre-dispersion was made, the suspensions were transferred to the test laboratory and vortexed approx. 10 seconds before 4 mL of the dispersion was added to each of the three of the reactors using a pipette, creating a theoretic starting concentration of 50 mg/L. The blank reactor was added 4 mL of the respective test medium. At selected time points; t_sampling_ = t_0_, t_1_, t_2_, t_4_, and t_24_ = 0, 1, 2, 4 and 24 hours, approx. 4 mL was collected from each reactor through the sampling septum using a spinal needle (Becton Dickinson, Madrid, Spain) and 5 mL plastic syringe (Henke Sass Wolf, Tuttlingen, Germany). The remaining particulate matter was immediately separated from dissolved ions using an Amicon Ultra-4 centrifugal filter with 3 kDa filter cut-off (product number Z740186, Merck, Darmstadt, Germany) and centrifuged at 4,400 × g, 4000 rpm, for 30 min using a Sorvall RC6+ centrifuge (Thermo Fisher Scientific, Waltham, USA). However, > 95 % is filtrated after 7 minutes, the filtration was continued for additional 23 min to ensure all was filtrated. It took approx. 2 min from finalizing the probe sonication, adding the particle suspension to the test reactors, until the first samples (t_0_) were collected and spinning in the centrifuge. After centrifugation, the filtrate was weighted to determine the actual sample size. To the filtered sample, 0.5 mL of 2% nitric acid (prepared in ultrapure water 18 MΩcm, acid obtained from Merck, Darmstadt, Germany) were added to stabilize the dissolved ions. The dissolved ionic fraction was analysed using inductively coupled plasma-mass spectrometry (ICP-MS).

*Phagolysosomal test fluid (PSF)*

Phagolysosomal simulant fluid (PSF) was prepared by dissolving the components of Table 1 in 2 L ultrapure water (18 MΩ.cm at 25°C) (Thermo Fisher Scientific, Waltham, USA). The solution was left overnight and filtered the following day through a polyvinylidene fluoride membrane 0.45 µm filter (Merck Millipore Ltd., Tullagreen, Ireland). PSF has a shelf-life of approx. 1-1.5 months stored at 5 °C protected from light. All chemicals were purchased from Merck (Darmstadt, Germany).

Table 1. Composition of phagolysosomal simulant fluid

| **Component** | **Chemical formula** | **Concentration [mg/L]** |
| --- | --- | --- |
| Sodium phosphate dibasic anhydrous | Na_2_HPO_4_ | 142 |
| Sodium chloride | NaCl | 6650 |
| Sodium sulphate anhydrous | Na_2_SO_4_ | 71 |
| Calcium chloride dihydrate | CaCl_2_·2H_2_O | 29 |
| Glycine | H_2_NCH_2_CO_2_H | 450 |
| Potassium hydrogen phthalate | (1-(HO_2_C)-2-(CO_2_K)-C_6_H_4_) | 4085 |
| Alkylbenzyldimethylammonium chloride | - | 50 |

*Dispersion of nanomaterials*

The test materials were dispersed in bovine serum albumin (BSA) - water after prewetting with ethanol (EtOH) following the NANOGENOTOX batch dispersion protocol validated as part of the FP7 NANoREG project. Before dispersion, a 0.05% w/v bovine serum albumin (BSA) solution was prepared in ultrapure water (18 MΩ.cm, 21 °C, Thermo Fisher Scientific, Waltham, USA). BSA (obtained from Sigma-Aldrich (now Merck), Darmstadt, Germany) was dissolved in ultrapure water to obtain a 1% w/v solution, stored overnight, and sterile-filtered (0.22 µm). The 1% w/v BSA solution was diluted to 0.05% w/v with ultrapure water.

Test material was weighted to a glass vial and pre-wetted with 75 µL 96% ethanol (Merck, Darmstadt, Germany) and dispersed with 0.05% w/v BSA solution to a final concentration of 2.56 mg/mL (or adjusted to 2.5 mg/mL for ease of dilution). A 400 W Branson Sonifier S-450D (Branson Ultrasonics Corp., Danbury, CT, USA) equipped with a 13 mm disruptor horn was used to sonicate the particle dispersion directly after adding the suspension media for 16 min with a 10% amplitude delivering a total acoustic energy of 7,056±0,103 kJ. The sonication was performed under constant cooling in an ice-water bath.

*Dynamic light scattering (DLS) and laser Doppler electrophoresis*

The pre-dispersions were measured for assessment of dispersion quality considering the mean hydrodynamic size (Z_ave_) and teh polydispersity index (PDI) of using a Malvern Zetasizer Nano ZS (Malvern Panalytics Ltd., Malvern, United Kingdom) device equipped with a 633 nm laser and using 173° as the measurement angle for non-invasive backscattering measurements. Measurements were completed in 700 µL disposable folded capillary cell (DTS1070, Malvern Panalytics) or standard disposable 1 mL polystyrene cuvettes and analysed after 5 min thermal equilibration. Measurements were conducted at 25 °C using the viscosity of water (0.8872 cP). Thermal equilibration was set to 120 seconds. For each sample, 10 consecutive measurements were made using automated optimization of measurement conditions. The results of these measurements are not reported here.

*Chemical analysis of supernatants*

The supernatants were analysed using inductively coupled plasma-mass spectrometry (ICP-MS) as a commercial service by Eurofins A/S. Elements are analysed with an expanded uncertainty of 10-15%.

*Plotting and statistical analysis*

Dissolution profiles and regression analysis were made using the Minitab^®^ 20 (Minitab, LLC.) Non-linear regression analyses were made displaying the 95% confidence interval and 95% predictive interval. Statistical significance was accepted at a P-value of 0.05 or lower.

Results

*Al_2_O_3_*

Figure 1 shows the results from the dissolution testing of Al_2_O_3_ and the associated regression curve of the dissolution profile. The results show rapid dissolution of the Al_2_O_3_ and relatively low solubility limit.


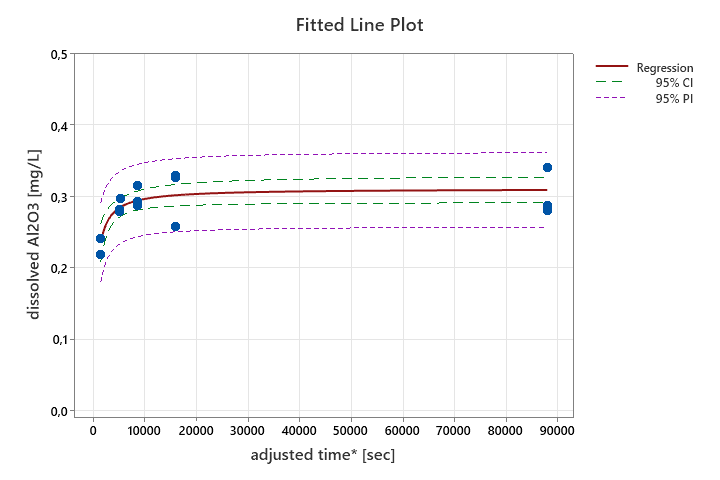


Figure 1. Dissolution profile and statistical regression curve for Al2O3 dissolution in PSF, plotted as function of time. The dissolved amount is calculated as Al_2_O_3_. 95% CI: Confidence interval at 95% confidence; PI: Predicted Interval at 95% confidence.

The statistical regression profile for Al_2_O_3_ dissolution is shown in Equation 1 and was calculated based on a a 2-parameter Mihaelis-Menton equation (Theta1 x X / Theta2 + X) in which Theta 1 gives the Y-axis asymptote (provides the estimated solubility limit; S_o_*) and Theta 2 displays the time where 50% of the material is dissolved.

Equation 1: dissolved Al_2_O_3_ [mg/L] = 0,311154 x t* / (480,403 + t*), where t* is given in seconds.

The regression statistics showed that the S_o_* was 0,311±0,009 mg Al_2_O_3_/L (95% CI: 0,293;0,330) and that the initial dissolution rate is relatively fast. According to the regression statistics, 50% of the added 50 mg/L material is dissolved after approximately 480 seconds (8 minutes). The amount of dissolved material at the starting time after 16 min predispersion by sonication in BSA-water was not determined, but may contribute significantly to the dissolved concentrations observed at the first sampling time-point. The initial dissolution rate cannot be determined experimentally, but may be derived from the regression curve as applied in [1,2]

*SnO_2_*

No dissolution of SnO_2_ was detected in PSF within the 24-hour time-period of the analysis.

References:

1. Holmfred E, Sloth JJ, Loeschner K, Jensen KA. Influence of Pre-Dispersion Media on the Batch Reactor Dissolution Behavior of Al2 O3 Coated TiO2 (NM-104) and Two ZnO (NM-110 and NM-111) Nanomaterials in Biologically Relevant Test Media. Nanomaterials. 2022;12.

2. Holmfred E, Loeschner K, Sloth JJ, Jensen KA. Validation and Demonstration of an Atmosphere-TemperaturepH-Controlled Stirred Batch Reactor System for Determination of (Nano)Material Solubility and Dissolution Kinetics in Physiological Simulant Lung Fluids. Nanomaterials. 2022;12.
