# Supplementary material for: Acute phase response following pulmonary exposure to soluble and insoluble metal oxide nanomaterials in mice
Source: Part Fibre Toxicol. 2023 Jan 17;20:4. doi: 10.1186/s12989-023-00514-0 (PMC9843849; doi:10.1186/s12989-023-00514-0)
Supplement: Supplementary file 2 — Additional file 2. Figure S1. Particle equivalent circular diameter distributions from TEM measurements on five metal oxide samples. [file 12989_2023_514_MOESM2_ESM.docx]

Additional information 2


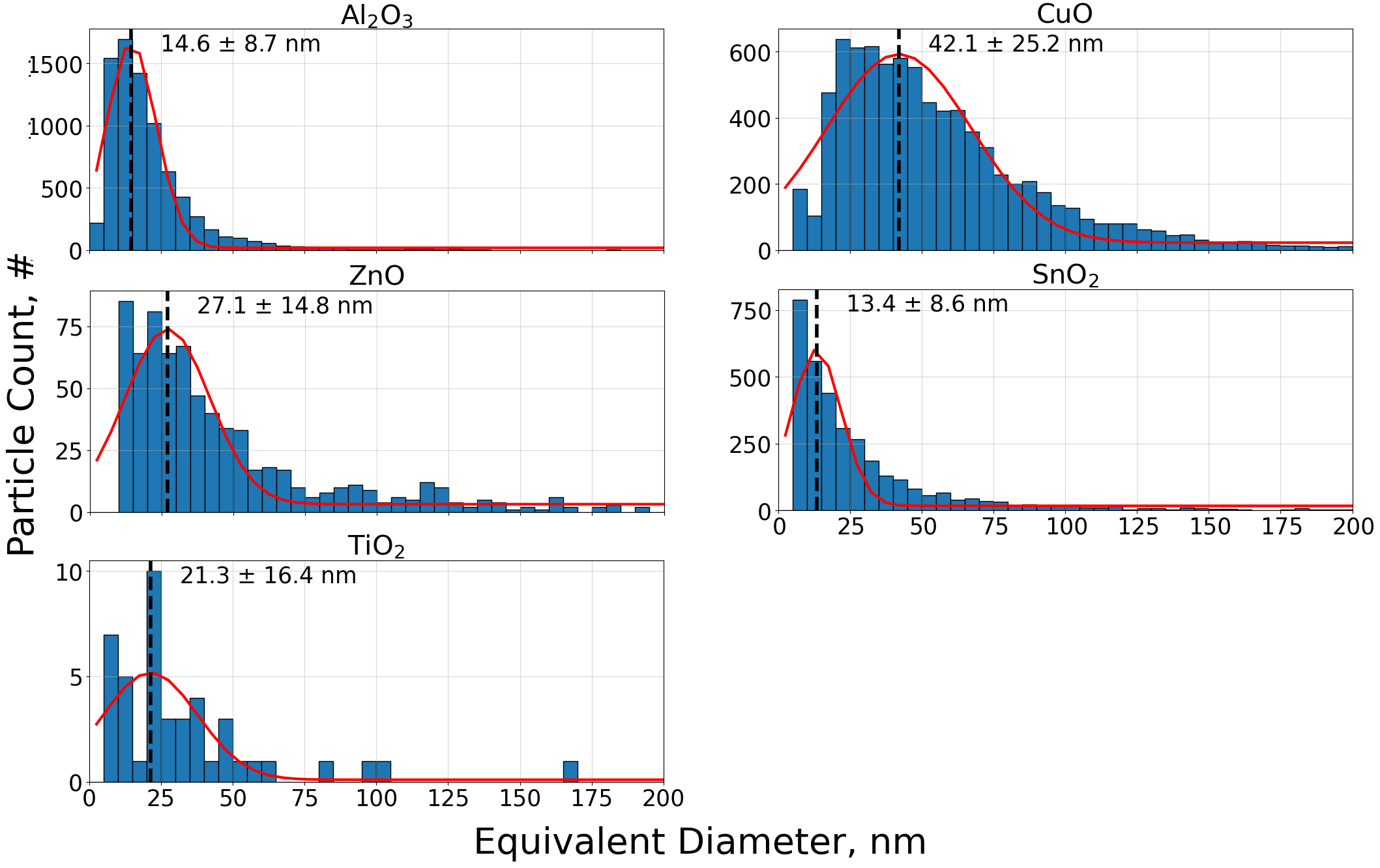


Figure S1. Particle equivalent circular diameter distributions from TEM measurements on five metal oxide samples. Samples were taken from the particle suspension in exposure vehicle. The distributions were fitted with a normal distribution, as seen in red, in order to get the mean size and standard deviation of the particle mode.
