# Supplementary material for: Acute phase response following pulmonary exposure to soluble and insoluble metal oxide nanomaterials in mice
Source: Part Fibre Toxicol. 2023 Jan 17;20:4. doi: 10.1186/s12989-023-00514-0 (PMC9843849; doi:10.1186/s12989-023-00514-0)
Supplement: Supplementary file 3 — Additional file 3. Table S1. Overview table of results from TEM analysis. [file 12989_2023_514_MOESM3_ESM.docx]

Additional information 3

Table S1. Overview table of results from TEM analysis.

| Sample | Images Analyzed, # | Area Analyzed, µm^2^ | Particles Analyzed, # | Mean agglomerate size, nm | Primary particle size, nm ^a^ | Lattice spacing, Å |
| --- | --- | --- | --- | --- | --- | --- |
| Al_2_O_3_ | 4 | 71.8 | 7920 | 15 ± 9 | 5.5 ± 5.5 | - |
| CuO | 6 | 2932 | 8239 | 42 ± 25 | 7.8 ± 6.8 | 2.7 ± 0.1 |
| SnO_2_ | 6 | 57 | 3438 | 13 ± 9 | 4.5 ± 2.3 | 3.4 ± 0.1 |
| TiO_2_ | 3 | 10 | 46 | 21 ± 16 | 6.6 ± 4.1 | 3.5 ± 0.1 |
| ZnO | 5 | 58 | 697 | 27 ± 15 | 5.7 ± 3.8 | 3.4 ± 0.1 |

Note: ^a^Primary particle size was measured for at least 15 different primary particles. Uncertainties are shown as the standard deviation of the measured quantities.
