# Supplementary material for: Acute phase response following pulmonary exposure to soluble and insoluble metal oxide nanomaterials in mice
Source: Part Fibre Toxicol. 2023 Jan 17;20:4. doi: 10.1186/s12989-023-00514-0 (PMC9843849; doi:10.1186/s12989-023-00514-0)
Supplement: Supplementary file 4 — Additional file 4. Figure S2. Number-based hydrodynamic size distributon of NMs dispersed in instillation vehicle measured by dynamic light scattering. [file 12989_2023_514_MOESM4_ESM.docx]

Additional information 4

Figure S2. Number-based hydrodynamic size distributions of nanomaterials dispersed in instillation vehicle measured by dynamic light scattering.
