# Supplementary material for: Acute phase response following pulmonary exposure to soluble and insoluble metal oxide nanomaterials in mice
Source: Part Fibre Toxicol. 2023 Jan 17;20:4. doi: 10.1186/s12989-023-00514-0 (PMC9843849; doi:10.1186/s12989-023-00514-0)
Supplement: Supplementary file 5 — Additional file 5. Figure S3. Mouse lung histopathology 28 days post-exposure to vehicle control, ZnO, CuO, Al2O3, SnO2, TiO2 and Printex 90. [file 12989_2023_514_MOESM5_ESM.docx]

Additional information 5


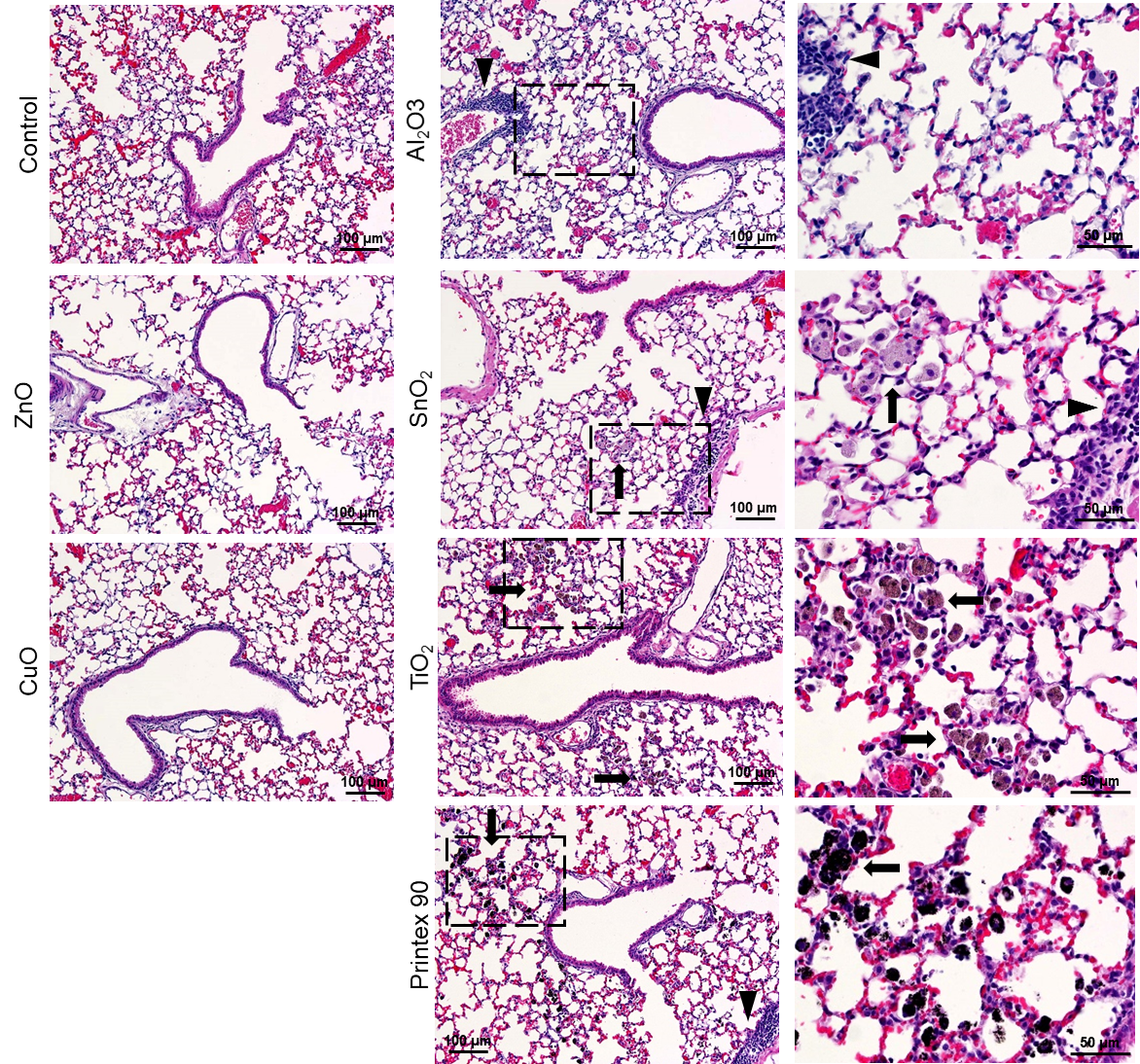


Figure S3. Mouse lung histopathology 28 days post-exposure to vehicle control, ZnO, CuO, Al_2_O_3_, SnO_2_, TiO_2_ and Printex 90. Macrophage aggregates in the alveolar region (arrows) and perivascular lymphocytic infiltration (arrowheads). Brightfield microscopy and H&E stain applies to all.
