# Supplementary material for: Acute phase response following pulmonary exposure to soluble and insoluble metal oxide nanomaterials in mice
Source: Part Fibre Toxicol. 2023 Jan 17;20:4. doi: 10.1186/s12989-023-00514-0 (PMC9843849; doi:10.1186/s12989-023-00514-0)
Supplement: Supplementary file 6 — Additional file 6. Table S2. Mean number and incidence of lymphocytic infiltrates and macrophage aggregates in lungs, 1 and 28 days post-exposure to NMs. [file 12989_2023_514_MOESM6_ESM.docx]

Additional information 6

Table S2. Mean number and incidence of lymphocytic infiltrates and macrophage aggregates in lungs, 1 and 28 days post-exposure to nanomaterials

| **Group** | **Day** | **Lymphocytic infiltrates^A^** | | **Macrophage aggregates^B^** | |
| --- | --- | --- | --- | --- | --- |
|  |  | **Mean**^C^ | **Incidence** | **Mean**^C^ | **Incidence** |
| Vehicle control | 1 | 0.0 | 0/6 | 0.0 | 0/6 |
|  | 28 | 0.1 | 1/8 | 0.0 | 0/8 |
| ZnO, 2 µg/mouse | 1 | 0.0 | 0/3 | 0.0 | 0/3 |
|  | 28 | 0.0 | 0/3 | 0.0 | 0/3 |
| CuO, 6 µg/mouse | 1 | 0.0 | 0/3 | 0.0 | 0/3 |
|  | 28 | 0.0 | 0/3 | 0.0 | 0/3 |
| CuO, 12 µg/mouse | 1 | 0.0 | 0/3 | 0.0 | 0/3 |
|  | 28 | 0.0 | 0/3 | 0.0 | 0/3 |
| Al_2_O_3_, 54 µg/mouse | 1 | 1.3 | 1/3 | 0.0 | 0/3 |
|  | 28 | 0.3 | 1/3 | 0.0 | 0/3 |
| SnO_2_, 162 µg/mouse | 1 | 0.7 | 1/3 | 0.0 | 0/3 |
|  | 28 | 0.7 | 2/3 | 0.7 | 2/3 |
| TiO_2_, 162 µg/mouse | 1 | 1.0^B^ | 2/3 | 0.0^D^ | 0/3^D^ |
|  | 28 | 0.3^B^ | 1/3 | 0.7 | 1/3 |
| Printex 90, 162 µg/mouse | 1 | 0.0 | 0/3 | 0.0 | 0/3 |
|  | 28 | 1.7 | 2/3 | 2.3 | 3/3 |

Notes:

The counting criteria are described in the materials and methods of the main manuscript.

^A^ Minimum 50 lymphocytes.

^B^ Minimum 5 macrophages in a group.

^C^ Mean number of infiltrates/aggregates per lung section with mosthistopathological changes.

^D^ Large infiltration of macrophages was observed 1 day post-exposure to TiO_2_ (mean 0.3, incidence 1/3). The size of macrophage aggregates was not quantified and therefore the severity is not fully represented in this table.
