# Supplementary material for: Acute phase response following pulmonary exposure to soluble and insoluble metal oxide nanomaterials in mice
Source: Part Fibre Toxicol. 2023 Jan 17;20:4. doi: 10.1186/s12989-023-00514-0 (PMC9843849; doi:10.1186/s12989-023-00514-0)
Supplement: Supplementary file 7 — Additional file 7. Table S3 and Table S4. Type and incidence of histological changes in livers from mice 28 days after intratracheal exposure to vehicle control or NMs. [file 12989_2023_514_MOESM7_ESM.docx]

Additional information 7

Table S3. Type and incidence of histological changes other than inflammatory cell infiltrations in livers from mice 28 days after intratracheal exposure to vehicle control or NMs.

| Change | Vehicle control | ZnO  2 µg/animal | CuO  6 µg/animal | CuO  12 µg/animal | Al_2_O_3_  54 µg/animal | SnO_2_  162 µg/animal | TiO_2_  162 µg/animal | Printex 90  162 µg/animal |
| --- | --- | --- | --- | --- | --- | --- | --- | --- |
| Mitosis | 8/18**^a^**  8/0/0/0/0**^b^** | 0/3 | 0/3 | 0/3 | 0/3 | 2/3  2/0/0/0/0 | 1/3  1/0/0/0/0 | 1/3  1/0/0/0/0 |
| Karyomegaly | 6/18  6/0/0/0/0 | 0/3 | 0/3 | 1/3  1/0/0/0/0 | 0/3 | 0/3 | 0/3 | 0/3 |
| Karyocytomegaly | 3/18 | 0/3 | 0/3 | 2/3 | 1/3 | 0/3 | 2/3 | 0/3 |
| Binucleate hepatocytes | 18/18  18/0/0/0/0 | 3/3  3/0/0/0/0 | 3/3  3/0/0/0/0 | 3/3  3/0/0/0/0 | 3/3  3/0/0/0/0 | 3/3  3/0/0/0/0 | 3/3  3/0/0/0/0 | 3/3  3/0/0/0/0 |
| Apparent increase in Kupffer cells | 2/18  2/0/0/0/0 | 1/3  1/0/0/0/0 | 0/3 | 2/3  2/0/0/0/0 | 1/3  1/0/0/0/0 | 1/3  1/0/0/0/0 | 0/3 | 0/3 |
| Kupffer cells with prominent nuclei | 0/3 | 0/3 | 0/3 | 1/3  1/0/0/0/0 | 0/3 | 0/3 | 0/3 | 0/3 |
| Hyperplasia of connective tissue near bile ductules or venules | 2/18 | 2/3  2/0/0/0/0 | 1/3  1/0/0/0/0 | 3/0/0/0/0 | 2/3  0/2/0/0/0 | 1/3  1/0/0/0/0 | 0/3 | 1/3  1/0/0/0/0 |
| Hyperplasia of oval cells | 1/18  1/0/0/0/0 | 1/3  1/0/0/0/0 | 0/3 | 0/3 | 1/3  1/0/0/0/0 | 0/3 | 0/3 | 0/3 |
| Apoptotic bodies in inflammatory cell infiltrates | 7/18 | 0/3 | 2/3 | 3/3 | 1/3 | 1/3 | 0/3 | 2/3 |
| Necrotic hepatocytes adjacent to inflammatory cell infiltrates | 5/18 | 2/3 | 0/3 | 2/3 | 1/3 | 2/3 | 2/3 | 1/3 |
| Congestion | 18/18 | 3/3 | 3/3 | 3/3 | 3/3 | 3/3 | 3/3 | 3/3 |
| Extravasation (blood cells in sinusoids) | 18/18 | 3/3 | 3/3 | 3/3 | 3/3 | 3/3 | 3/3 | 3/3 |
| Vacuolization of cytoplasm of hepatocytes (midzonal, physiological state) | 18/18 | 3/3 | 3/3 | 3/3 | 3/3 | 3/3 | 3/3 | 3/3 |

Notes:

**^a^**: Incidence of each change is expressed by the number of animals with a given change of a total animals examined in the group. The livers were obtained from all (n=18) vehicle control mice and from 3 high-dose exposed mice in each test group.

**^b^**: Severity of a given change was evaluated in a semi-quantitative way using a five grade scoring system. Grade 1: minimal/very few/very small; grade 2: mild/few/small; grade 3: moderate/moderate number/moderate size; grade 4: marked/many/large; grade 5: massive/extensive number/extensive size. Results for severity are presented as a number of animals per group for which a given change was assigned to a certain grade either G1/G2/G3/G4/G5 e.g. the severity reported as 3/0/0/0/0 means that 3 animals in the group had a given change of the severity graded as G1 (minimal).

Table S4. Incidence and number of inflammatory cell infiltrations in the livers from mice 28 days after intratracheal instillation with vehicle control or NMs.

| Inflammatory cell infiltration | Vehicle control | ZnO  2 µg/animal | CuO  6 µg/animal | CuO  12 µg/animal | Al_2_O_3_  54 µg/animal | SnO_2_  162 µg/animal | TiO_2_  162 µg/animal | Printex 90  162 µg/animal |
| --- | --- | --- | --- | --- | --- | --- | --- | --- |
| All (big and small) | |  |  |  |  |  |  |  |
| Incidence^a^ | 16/18 | 3/3 | 3/3 | 3/3 | 3/3 | 3/3 | 3/3 | 2/3 |
| Total^b^ | 45 | 5 | 12 | 13 | 5 | 22 | 8 | 8 |
| Mean^c^ | 2.5 | 1.7 | 4.0 | 4.3 | 1.7 | 7.3 | 2.7 | 2.7 |
| (SD) | (1.9) | (0.7) | (3.5) | (4.2) | (0.7) | (10.1) | 1.2 | (2.3) |
| Multiplicity^d^ | 2.8 | 1.7 | 4.0 | 4.3 | 1.7 | 7.3 | 2.7 | 4.0 |
|  |  |  |  |  |  |  |  |  |
| Small |  |  |  |  |  |  |  |  |
| Incidence^a^ | 12/18 | 2/3 | 2/3 | 3/3 | 2/3 | 3/3 | 1/3 | 2/3 |
| Total^b^ | 27 | 4 | 9 | 9 | 2 | 13 | 2 | 4 |
| Mean^c^ | 1.5 | 1.3 | 3.0 | 3.0 | 0.7 | 4.3 | 0.7 | 1.3 |
| (SD) | (1.4) | (1.2) | (4.4) | (2.0) | (0.6) | (5.8) | (1.2) | (1.2) |
| Multiplicity^d^ | 2.3 | 2.0 | 4.5 | 3.0 | 1 | 4.3 | 2 | 2.0 |
|  |  |  |  |  |  |  |  |  |
| Big |  |  |  |  |  |  |  |  |
| Incidence^a^ | 11/18 | 1/3 | 2/3 | 2/3 | 3/3 | 2/3 | 3/3 | 2/3 |
| Total^b^ | 18 | 1 | 3 | 4 | 3 | 9 | 6 | 4 |
| Mean^c^ | 1.0 | 0.3 | 1.0 | 1.3 | 1.0 | 3.0 | 2.0 | 1.3 |
| (SD) | (1.1) | (0.6) | (1.0) | (1.5) | (0.0) | (4.4) | 0.0 | (1.2) |
| Multiplicity^d^ | 1.6 | 1.0 | 1.5 | 2.0 | 1.0 | 4.5 | 2.0 | 2.0 |

Notes:

^a^: Incidence is expressed by the number of livers with one or more inflammatory cell infiltrations of total number examined in the group.

^b^: Number of inflammatory cell infiltrations of a given type from all liver samples examined in the group. For each animal, a liver sample from the left lobe was used for counting of inflammatory cell infiltrations.

^c^: Mean number of inflammatory cell infiltrations small or big/number of liver samples examined.

^d^: Mean number of inflammatory cell infiltrations of a given type per group/numbers of liver samples with the given inflammatory cell infiltration in the group.
