# Supplementary material for: Acute phase response following pulmonary exposure to soluble and insoluble metal oxide nanomaterials in mice
Source: Part Fibre Toxicol. 2023 Jan 17;20:4. doi: 10.1186/s12989-023-00514-0 (PMC9843849; doi:10.1186/s12989-023-00514-0)
Supplement: Supplementary file 8 — Additional file 8. Table S5. BAL cell count 1 and 28 days after exposure to NMs. [file 12989_2023_514_MOESM8_ESM.docx]

Additional information 8

Table S5. BAL cell count 1 and 28 days after exposure to nanomaterials.

|  | **Vehicle control** | **ZnO** | | **CuO** | | |
| --- | --- | --- | --- | --- | --- | --- |
|  | **0 µg** | **0.7 µg** | **2 µg** | **2 µg** | **6 µg** | **12 µg** |
| 1 day |  |  |  |  |  |  |
| Total BAL cells (x 10^3^) | 54.9 ± 17.2 | 49.6 ± 16.6 | 50.9 ± 19.7 | 45.5 ± 15.2 | 58.2 ± 24.5 | 37.1 ± 8.4 |
| Neutrophils(x 10^3^) | 3.5 ± 2.7 | 13.0 ± 13.1 | 7.8 ± 9.7 | 11.4 ± 5.9 | 20.8 ± 13.5 | 10.6 ± 6.3 |
| Macrophages (x 10^3^) | 47.9 ± 19.1 | 34.0 ± 18.0 | 41.3 ± 16.1 | 29.4 ± 8.8 | 33.1 ± 10.4 | 23.8 ± 7.5 |
| Eosinophils (x 10^3^) | 0.4 ± 1.0 | 0.4 ± 0.6 | 0.3 ± 0.2 | 1.8 ± 3.1 | 0.8 ± 0.9 | 0.3 ± 0.8 |
| Lymphocytes (x 10^3^) | 0.9 ± 0.9 | 2.0 ± 0.8 | 1.6 ± 0.6 | 1.5 ± 0.7 | 2.5 ± 1.7 | 2.3 ± 1.8 |
| Epithelial (x 10^3^) | 0.4 ± 1.0 | 0.1 ± 0.3 | 0.0 ± 0.0 | 1.5 ± 0.7 | 1.1 ± 1.2 | 0.0 ± 0.0 |
|  |  |  |  |  |  |  |
| 28 days |  |  |  |  |  |  |
| Total BAL cells (x 10^3^) | 47.0 ± 21.6 | 38.5 ± 8.0 | 47.2 ± 11.3 | 58.1 ± 23.5 | 62.8 ± 26.9 | 50.2 ± 5.2 |
| Neutrophils(x 10^3^) | 0.7 ± 0.8 | 0.8 ± 0.4 | 0.5 ± 0.3 | 0.5 ± 0.5 | 2.0 ± 3.4 | 0.2 ± 0.3 |
| Macrophages (x 10^3^) | 43.4 ± 19.5 | 36.5 ± 8.5 | 44.2 ± 12.7 | 55.1 ± 22.3 | 58.1 ± 24.3 | 48.0 ± 5.8 |
| Eosinophils (x 10^3^) | 0.7 ± 2.5 | 0.0 ± 0.1 | 0.1 ± 0.1 | 0.0 ± 0.1 | 0.5 ± 1.2 | 0.4 ± 0.6 |
| Lymphocytes (x 10^3^) | 2.0 ± 2.7 | 1.2 ± 0.6 | 1.8 ± 1.0 | 1.0 ± 0.5 | 1.5 ± 1.3 | 1.6 ± 1.2 |
| Epithelial (x 10^3^) | 0.3 ± 0.5 | 0.0 ± 0.0 | 0.6 ± 1.5 | 1.4 ± 1.4 | 0.7 ± 0.7 | 0.0 ± 0.0 |
|  |  |  |  |  |  |  |
|  | **Al_2_O_3_** | | **SnO_2_** | | **TiO_2_** | **Printex 90** |
|  | **18 µg** | **54 µg** | **54 µg** | **162 µg** | **162 µg** | **162 µg** |
| 1 day |  |  |  |  |  |  |
| Total BAL cells (x 10^3^) | 44.2 ± 8.2 | 75.3 ± 15.3 | 64.2 ± 22.4 | 160.7 ± 55.2 | 196.5 ± 68.1 | 152.3 ± 34.7 |
| Neutrophils(x 10^3^) | 2.6 ± 2.6 | 26.2 ± 8.7 | 10.4 ± 13.5 | 118.4 ± 52.0 | 142.4 ± 58.4 | 115.2 ± 19.1 |
| Macrophages (x 10^3^) | 40.8 ± 6.2 | 45.1 ± 15.3 | 50.6 ± 14.3 | 34.7 ± 9.4 | 40.3 ± 19.9 | 16.6 ± 9.2 |
| Eosinophils (x 10^3^) | 0.1 ± 0.1 | 2.4 ± 1.8 | 1.3 ± 2.9 | 4.3 ± 5.0 | 11.1 ± 10.5 | 17.7 ± 21.1 |
| Lymphocytes (x 10^3^) | 0.7 ± 0.4 | 1.7 ± 0.5 | 0.5 ± 0.4 | 1.9 ± 2.5 | 2.4 ± 1.2 | 2.4 ± 1.5 |
| Epithelial (x 10^3^) | 0.0 ± 0.1 | 0.0 ± 0.0 | 1.3 ± 1.1 | 1.3 ± 1.2 | 0.3 ± 0.5 | 0.5 ± 0.6 |
|  |  |  |  |  |  |  |
| 28 days |  |  |  |  |  |  |
| Total BAL cells (x 10^3^) | 41.5 ± 12.5 | 73.6 ± 55.7 | 51.5 ± 12.0 | 84.2 ± 26.2 | 87.8 ± 41.4 | 97.0 ± 37.6 |
| Neutrophils(x 10^3^) | 0.9 ± 0.6 | 3.2 ± 2.4 | 1.3 ± 2.4 | 4.5 ± 3.3 | 5.9 ± 7.0 | 22.5 ± 15.4 |
| Macrophages (x 10^3^) | 38.2 ± 12.3 | 67.7 ± 53.2 | 47.8 ± 9.4 | 64.3 ± 18.4 | 69.1 ± 29.7 | 58.8 ± 20.6 |
| Eosinophils (x 10^3^) | 1.2 ± 2.7 | 0.1 ± 0.2 | 0.0 ± 0.0 | 0.1 ± 0.3 | 0.1 ± 0.4 | 0.1 ± 0.2 |
| Lymphocytes (x 10^3^) | 1.2 ± 1.0 | 2.6 ± 1.5 | 1.9 ± 1.1 | 14.4 ± 8.9 | 12.3 ± 8.0 | 15.4 ± 4.2 |
| Epithelial (x 10^3^) | 0.0 ± 0.0 | 0.0 ± 0.0 | 0.6 ± 0.8 | 0.9 ± 0.5 | 0.3 ± 0.7 | 0.3 ± 0.3 |
