# Supplementary material for: Acute phase response following pulmonary exposure to soluble and insoluble metal oxide nanomaterials in mice
Source: Part Fibre Toxicol. 2023 Jan 17;20:4. doi: 10.1186/s12989-023-00514-0 (PMC9843849; doi:10.1186/s12989-023-00514-0)
Supplement: Supplementary file 9 — Additional file 9. Table S6. Publications used in the correlation analysis. [file 12989_2023_514_MOESM9_ESM.docx]

Additional information

Table S6. Publications used in the correlation analysis.

| **Publication** | **Nanomaterials** |
| --- | --- |
| Danielsen et al. (2020) | TiO_2_ |
| Billing et al. (2020) | Co_3_O_4_ |
|  | Fe_3_O_4_ |
|  | Carbon black (Printex 90) |
| Hadrup et al. (2021) | CuO |
|  | Carbon black (Printex 90) |
| Saber et al. (2019) | TiO_2_ |
| Jacobsen et al. (2015) | ZnO |
|  | Carbon black (Printex 90) |
| Jacobsen et al. (2009) | Carbon black (Printex 90) |
| Hadrup et al. (2019) | ZnO |
|  | Carbon black (Printex 90) |
| Halappanavar et al. (2015) | TiO_2_ |
| Wallin et al. (2017) | TiO_2_ |
| Hadrup et al. (2020) | Fe_2_O_3_ |
|  | Carbon black (Printex 90) |
| Saber et al. (2012) | TiO_2_ |
| Bourdon et al. (2012) | Carbon black (Printex 90) |
