# Supplementary material for: Acute phase response following pulmonary exposure to soluble and insoluble metal oxide nanomaterials in mice
Source: Part Fibre Toxicol. 2023 Jan 17;20:4. doi: 10.1186/s12989-023-00514-0 (PMC9843849; doi:10.1186/s12989-023-00514-0)
Supplement: Supplementary file 10 — Additional file 10. Figures S4 and S5. Saa3 mRNA levels in lung tissue and SAA3 protein levels in plasma from mice, 1 day after intratracheally instillation with 2, 6 or 18 µg of ZnO. [file 12989_2023_514_MOESM10_ESM.docx]

Additional information 10

Figure S4. *Saa3* mRNA levels in lung tissue from mice, 1 day after intratracheally instillation with 2, 6 or 18 µg of ZnO. Data are shown as mean and bars represent SD. More details can be found in Jacobsen et al. (2015).

Figure S5. SAA3 protein levels in plasma from mice, 1 day after intratracheally instillation with 6 or 18 µg of ZnO. Data are shown as mean and bars represent SD. More details can be found in Jacobsen et al. (2015).
