# Supplementary material for: Acute phase response following pulmonary exposure to soluble and insoluble metal oxide nanomaterials in mice
Source: Part Fibre Toxicol. 2023 Jan 17;20:4. doi: 10.1186/s12989-023-00514-0 (PMC9843849; doi:10.1186/s12989-023-00514-0)
Supplement: Supplementary file 11 — Additional file 11. Figures S6, S7, S8 and S9. Correlations between dosed mass and neutrophil numbers, Saa3 mRNA levels, Saa1 mRNA levels, SAA3 plasma protein levels or SAA1/2 plasma protein levels, and neutrophil number from vehicle control and Printex 90 from studies used in correlations. [file 12989_2023_514_MOESM11_ESM.docx]

Additional information 11

Figure S6. Correlations between dosed mass and neutrophil numbers, 1 (A) and 28 (B) days after exposure, and dosed mass and *Saa3* mRNA levels in lung tissue, 1 (C) and 28 (D) days after exposure. Results from the present study were combined with previously published data [14,17,30–34,36,37,45,53,97].

Figure S7. Correlations between dosed mass and *Saa1* mRNA levels in liver tissue, 1 (A) and 28 (B) days after exposure, and between dosed mass and SAA3 (C) and SAA1/2 (D) protein in plasma, in 1 day after exposure [17,30].

Figure S8. Correlations between ZnO and CuO dosed mass and: (A) neutrophil numbers, and (B) *Saa3* mRNA levels in liver tissue. All data considers 28 day after exposure to either ZnO or CuO. Results from the present study were combined with previously published data [14,30,37].

Figure S9. Neutrophil number 1 day post-exposure to vehicle control and 162 µg/mouse Printex 90 from studies used for correlations. (A) [36,38]; (B) [37,53]; (C) [14,33,47]; (D) [31]; (E) [32]; (F) [30]; and (G) the present study.
